# Site-Specific Phosphorylation of the DNA Damage Response Mediator Rad9 by Cyclin-Dependent Kinases Regulates Activation of Checkpoint Kinase 1

**DOI:** 10.1371/journal.pgen.1003310

**Published:** 2013-04-04

**Authors:** Carla Manuela Abreu, Ramesh Kumar, Danielle Hamilton, Andrew William Dawdy, Kevin Creavin, Sarah Eivers, Karen Finn, Jeremy Lynn Balsbaugh, Rosemary O'Connor, Patrick A. Kiely, Jeffrey Shabanowitz, Donald F. Hunt, Muriel Grenon, Noel Francis Lowndes

**Affiliations:** 1 Centre for Chromosome Biology, School of Natural Science, National University of Ireland Galway, Galway, Ireland; 2 Department of Chemistry, University of Virginia, Charlottesville, Virginia, United States of America; 3 Cell Biology Laboratory, Department of Biochemistry, BioSciences Institute, University College Cork, Cork, Ireland; 4 Department of Life Sciences, and Materials and Surface Science Institute, University of Limerick, Limerick, Ireland; Newcastle University, United Kingdom

## Abstract

The mediators of the DNA damage response (DDR) are highly phosphorylated by kinases that control cell proliferation, but little is known about the role of this regulation. Here we show that cell cycle phosphorylation of the prototypical DDR mediator *Saccharomyces cerevisiae* Rad9 depends on cyclin-dependent kinase (CDK) complexes. We find that a specific G2/M form of Cdc28 can phosphorylate *in vitro* the N-terminal region of Rad9 on nine consensus CDK phosphorylation sites. We show that the integrity of CDK consensus sites and the activity of Cdc28 are required for both the activation of the Chk1 checkpoint kinase and its interaction with Rad9. We have identified T125 and T143 as important residues in Rad9 for this Rad9/Chk1 interaction. Phosphorylation of T143 is the most important feature promoting Rad9/Chk1 interaction, while the much more abundant phosphorylation of the neighbouring T125 residue impedes the Rad9/Chk1 interaction. We suggest a novel model for Chk1 activation where Cdc28 regulates the constitutive interaction of Rad9 and Chk1. The Rad9/Chk1 complex is then recruited at sites of DNA damage where activation of Chk1 requires additional DDR–specific protein kinases.

## Author Summary

Human cells activate the DNA damage response (DDR) to repair DNA damage and to prevent cells with DNA damage from proliferating. Alterations to the DDR are strongly implicated in the development of cancer. Using the budding yeast model system, we have studied how the regulation of the key DDR component Rad9 is integrated into cell cycle control. The cyclin-dependent kinase Cdc28 that regulates the yeast cell cycle also extensively phosphorylates Rad9 during cell cycle progression. We show here that Cdc28 controls Rad9 function in the activation of the important downstream DNA damage effector kinase Chk1. Two sites of phosphorylation in the N-terminus of Rad9 are crucial for the physical interaction between Rad9 and Chk1 regulated by Cdc28. We propose a novel model for Chk1 activation whereby a subset of Rad9 and Chk1 interacts constitutively in the absence of DNA damage. The Rad9/Chk1 complex is recruited to sites of DNA damage where activation of Chk1 involves additional DDR–specific protein kinases. Human cells contain multiple Rad9-like proteins that are also known to be cell cycle phosphorylated in the absence of exogenous DNA damage, suggesting that our observations may have important implications for DDR regulation in human cells.

## Introduction

Eukaryotic cells have developed highly conserved surveillance pathways known as the DNA damage response (DDR) to preserve genome integrity after genotoxic insult. These pathways inhibit replication and segregation of damaged DNA by activating checkpoints and regulating transcription, replication and repair [1]. Defects in the DDR contribute to human cancer primarily due to defective induction of apoptosis and senescence [2].

Central to the DDR are protein kinases that are activated by DNA lesions. The human phosphatidylinositol 3-kinase-like kinases (PIKKs), ATM, ATR and DNA-PK occupy central points in the DNA damage-induced signalling pathways [1], [3]. ATM corresponds to Tel1, and ATR corresponds to Mec1 in *Saccharomyces cerevisiae* and Rad3 in *Schizosaccharomyces pombe* [3] ; DNA-PK does not exist in the budding and fission yeast model systems. Once activated by DNA-protein structures generated in response to lesions, PIKKs regulate numerous DDR proteins including the downstream ‘checkpoint’ kinases Chk1 (in all three species) and Chk2 (Rad53 or Cds1 in budding and fission yeast respectively). These two ‘effector’ kinases in turn phosphorylate target proteins leading to the downstream biological consequences of DDR activation [4].

DNA damage response mediators contribute to the PIKK-dependent activation of checkpoint kinases by acting as molecular adaptors that facilitate protein-protein interactions at sites of DNA damage [5]–[7]. The first checkpoint protein identified was *S. cerevisiae* Rad9 [8] and this is the prototypical DDR mediator. Rad9 is a 148 kDa protein required for cell survival in response to DNA damage. It is homologous to *S. pombe* Crb2 [9], [10] and shares functional and structural similarities with three human mediators 53BP1, MDC1 and BRCA1 [5]–[7]. Rad9 is required throughout the cell cycle for checkpoint delays [11], but also has other functions in the DDR including roles in DNA repair [12]–[15].

Mediators are typically phosphoproteins that are modified by multiple kinases including the PIKKs as a result of DNA damage [9], [16]–[22]. DNA damage-induced and PIKK-dependent phosphorylation of budding yeast Rad9 is required for its oligomerisation [23] and activation. PIKK-dependent Rad9 phosphorylation occurs once the mediator is recruited to the damaged chromatin by either of two redundant recruitment pathways [24]. One is dependent on the interaction of Rad9 with two histone modifications and the other is independent of these modifications but involves Rad9 interaction with the Dpb11 mediator. DNA damage-induced Rad9 phosphorylation correlates with the remodeling of a ≥850 kDa Rad9 complex into a smaller 560 kDa complex containing the DNA damage-induced hyperphosphorylated form of Rad9 [25], [26]. This mediates Rad9 function as an adaptor-catalyst for activation of the Rad53 kinase [25]–[27]: Rad9 is hyperphosphorylated by PIKKs once recruited to sites of DNA damage and this generates a recruitment surface for Rad53, which in turn is also phosphorylated by PIKKs to become pre-activated. Full activation of Rad53 requires its in *trans* autophosphorylation, catalyzed by the increased local concentration of Rad53 at the Rad9 scaffold [27].

Chk1 activation is well conserved from yeast to human. PIKK-dependent phosphorylation of its C-terminal region allows Chk1 to switch from an inactive to an active state in response to DNA damage [28], [29]. This is followed by in *cis* autophosphorylation on the same C-terminal region [30], which is presumed to be important for the propagation of the human CHK1 signal in the nucleus [30]. Chk1 in *cis* autophosphorylation upon DNA damage is also conserved in budding yeast [31] suggesting that it could have the same function as its human counterpart.

ATR phosphorylation of human CHK1 depends on a variety of DNA damage mediators [4]. Of these, claspin-dependent activation of CHK1 is the best characterised and involves PIKK-dependent phosphorylation of claspin itself [32], [33]. The orthologue of claspin in both budding and fission yeast is Mrc1 although in the DNA replication checkpoint budding yeast Mrc1 primarily regulates Rad53, rather than Chk1 [34]. In both yeast models the Rad9/Crb2 mediator proteins have been mostly implicated in Chk1 activation after DNA damage through their scaffolding role. Their N-terminal region is also known to be important for Chk1 activation although the specific mechanism is not well characterised [35], [36]. Rad9-like mediator proteins in higher cells, including BRCA1 and MDC1, play complex roles in CHK1 activation that are not entirely understood [4], [32].

DDR induction after DNA double-strand breaks (DSBs) involves cyclin-dependent kinases (CDKs) in addition to the recognition and processing of the breaks [37]. CDK activity controls the generation of single-strand DNA, leading to the amplification of the checkpoint signal by stimulating the switch from ATM to ATR activity, as well as impacting upon the choice of DSB repair mechanisms [4], [37]. CDK activity might also regulate other DDR proteins not necessarily involved at the DSB processing step [4], [37]. For example, CDK activity regulates multiple steps of the homologous recombination process [37].

DNA damage mediators are frequently phosphorylated during cell cycle progression in the absence of exogenous DNA damage [19], [20], [38]–[41]. The ‘Rad9-like’ mediators share a large number of consensus motifs for phosphorylation by CDKs (S/T-P-x-K/R or minimal S/T-P sites) but their functions remain unknown. Rad9 contains an exceptionally high density of CDK motifs with twenty S/T-P sites of which nine conform to the full consensus [42], [43]. In undamaged cells the mobility of Rad9 during electrophoresis suggests that it is heavily modified. Phosphatase-sensitive modified forms with slow mobility in SDS PAGE have been observed in S and M phase-arrested cells [20]. Not surprisingly, Rad9 has been identified as a CDK substrate *in vitro* [43], [44]. To date mass spectrometric studies support the *in vivo* phosphorylation of 15 of these CDK consensus sites [45]–[47], although the biological role of CDK-dependent phosphorylation of Rad9 is not well characterised.

Here we have investigated the phosphorylation of Rad9 during the S, G2 and M phases of the cell cycle and found that it is mediated by the Cdc28/Clb complexes. Cdc28 is the *S. cerevisiae* counterpart of human CDK1, and can bind six different cyclin B equivalents, Clb1-6. We show that the N-terminal region of Rad9 containing 9 consensus CDK phosphorylation sites is specifically phosphorylated *in vitro* by Cdc28/Clb2. Mutational analyses indicate that the N-terminal consensus CDK sites in Rad9 regulate a DNA damage-independent interaction between Chk1 and Rad9, as well as the activation of Chk1-dependent but Rad53-independent, signalling after DNA damage. We show that two CDK sites T125 and T143 are of particular importance for Rad9-dependent Chk1 activation. Our results suggest that phosphorylation of T143 stimulates Rad9 interaction with Chk1, whereas phosphorylation of T125 is inhibitory. We present a model in which prior CDK-dependent phosphorylation of the Rad9 N-terminus during S, G2 and M phases is required for the interaction between Chk1 and Rad9. PIK kinase-dependent phosphorylation of both Chk1 and Rad9 at sites of DNA damage is then followed by Chk1 activation and its release from site of DNA damage simultaneously with Rad9.

## Results

### DNA damage-independent phosphorylation of Rad9 during S, G2, and M requires Cdc28/Clb activity

Rad9 is phosphorylated in the absence of DNA damage ([11], [17], [20] and Figure S1), and as a result it migrates in SDS-PAGE with an apparent mobility ranging from 180 to 220 kDa [20]. These Rad9 phospho-forms found in normally cycling cells have faster mobility [48] than the slowest Mec1/Tel1-dependent migrating forms of Rad9 observed in response to DNA damage [11], [17], [20], [48]. To more clearly distinguish between the DNA damage and cell cycle regulated forms of Rad9, here we term the former as D-Rad9 and the latter as C-Rad9. C-Rad9, previously termed hypo-phosphorylated Rad9, was originally detected in S or M, but not G1, arrested cells [20].

**Figure 1:**
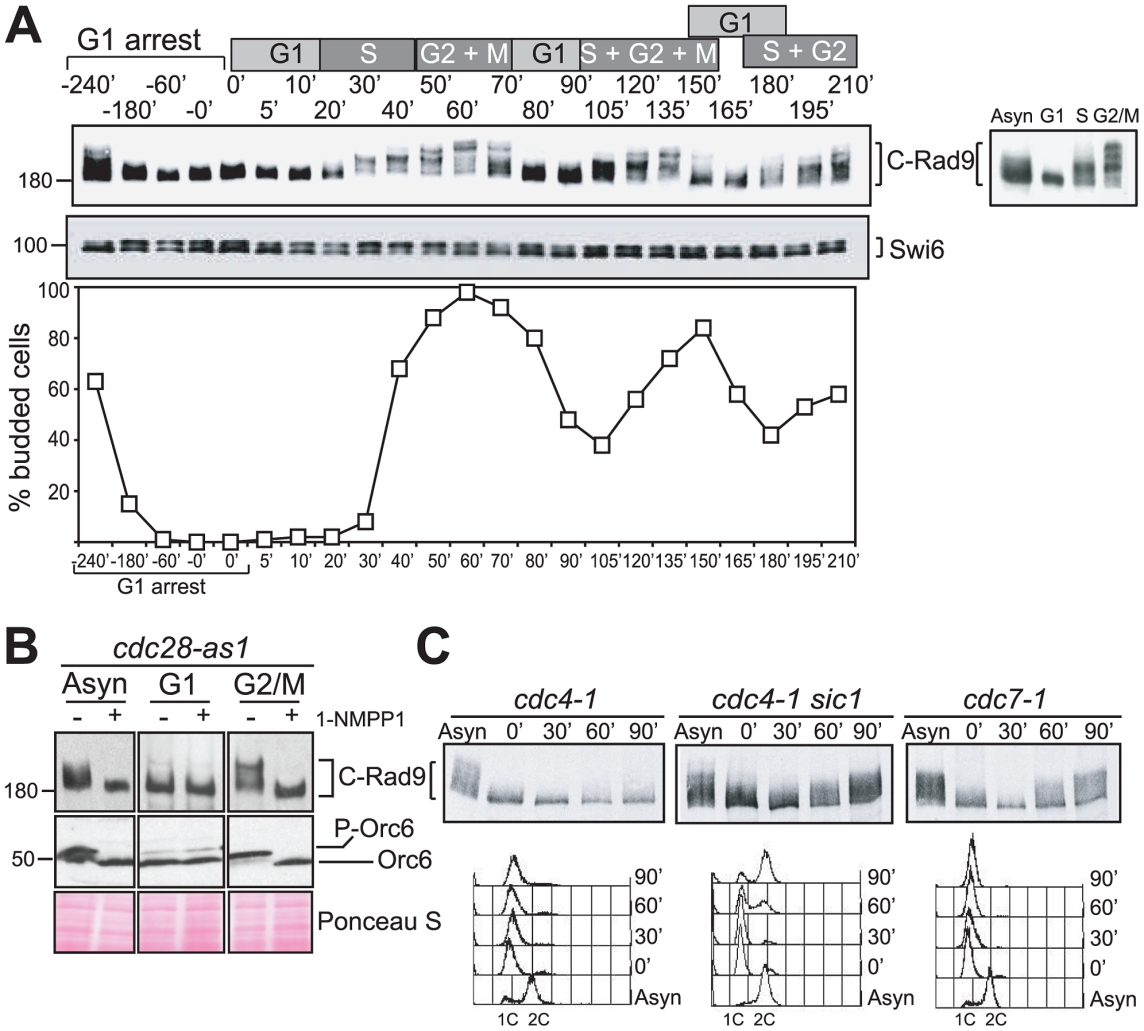
Rad9 is phosphorylated by Cdc28 in the absence of DNA damage. (A) Cell cycle regulation of phosphorylated forms of Rad9. An α-factor block and release experiment using yeast strain CG378. Rad9 phospho-forms were identified in protein extracts by western blotting. The phospho-protein Swi6 serves as a loading control. The cell cycle phases of synchronously cycling cells determined by budding analysis are indicated above the blots. The insert in the graph shows Rad9 from asynchronously growing cells or cells arrested with α-factor, HU or nocodazole. (B) Rad9 phosphorylation is dependent on Cdc28 activity. Rad9 and Orc6 western blots performed with extracts prepared from asynchronously growing, α-factor (G1) or nocodazole (G2/M) arrested *cdc28-as1* cells either mock or 1-NMPP1 treated. (C) Rad9 phosphorylation is dependent on Cdc28/Clb activity. The indicated cells were arrested with α-factor, shifted to 30°C and samples were taken at the indicated times for Rad9 western and FACS analysis. See also Figure S1 for related experiments.

To exclude the possibility that C-Rad9 phosphorylation forms were mainly induced in response to cell cycle arrest treatment, we determined the phosphorylation profile of Rad9 during normal cell cycle progression in an arrest and release experiment (Figure 1A). The fastest migrating C-Rad9 forms were detected in cells progressing through G1 phase, with slower migrating C-Rad9 forms appearing as cells entered S phase and persisting with ever decreasing mobility as cells moved through S, G2 and into mitosis (Figure 1A). These slower forms disappeared towards the end of mitosis, with the fastest migrating form of C-Rad9 reappearing in G1 cells in a pattern that can be observed over at least 2.5 cell cycles. This indicates that C-Rad9 phosphorylation occurs during normal cell proliferation from S to M phases in the absence of DNA damage and not only in S or M phase-induced cell cycle arrests. Interestingly, the cyclic pattern of Rad9 phosphorylation directly mirrors the cell cycle activity of budding yeast Cdc28, which regulates cell cycle progression by association with 9 different cyclins [49]. The Cdc28/Cln1-3 complexes are active in G1 phase and have lower kinase activity than the Cdc28/Clb1-6 complexes that function specifically in S, G2 and mitosis [49]. Interestingly, Rad9 was identified as one of 360 proteins to be phosphorylated *in vitro* by Cdc28/Clb2 and Cdc28/Clb5 complexes [43], [44]. We hypothesised that Rad9 is increasingly phosphorylated during passage through S, G2 and M phases of the cell cycle, and that the Cdc28/Clbs complexes are responsible for Rad9 cell cycle phosphorylation profile *in vivo*.

We first tested the hypothesis that the *in vivo* phosphorylated forms of Rad9 observed during cell cycle progression require Cdc28 activity. We used cells expressing Cdc28-as1, an analogue-sensitive mutant of Cdc28 [50] in which Cdc28-as1 is inactivated within 5–15 minutes in the presence of the bulky non-hydrolysable ATP analogue 1-NMPP1. Dephosphorylation of Cdc28 substrates under these conditions is then dependent upon counteracting phosphatases [43]. We followed inactivation of Cdc28-as1 [43] by monitoring dephosphorylation of Orc6, a known Cdc28 target [51]. The slower migrating forms of C-Rad9 detected in asynchronous and nocodazole-arrested G2/M cells were completely abolished as a consequence of this Cdc28 inactivation (Figure 1B). The migration of Rad9 in G1 arrested cells was not affected by the inactivation of Cdc28. Thus, the increasing phosphorylation of Rad9 detectable as decreased electrophoretic mobility of Rad9 when cells progress through S, G2 and M phase is dependent upon Cdc28.

The B type cyclins Clb1-6 are candidate cyclin partners of Cdc28 required for Rad9 cell cycle phosphorylation. We therefore examined C-Rad9 gel mobility in temperature sensitive mutants with different levels of Cdc28/Clb activity at the non-permissive temperature (Figure 1C). Cdc4 is an F-box protein of the SCF E3 ubiquitin ligase complex that determines substrate specificity and is required for destruction of Sic1, a potent inhibitor of CDK complexes containing B-type cyclins [49]. We generated G1 cells with high levels of Sic1 by arresting *cdc4-1* cells with α-factor and shifting to the restrictive temperature [52]–[54] (Figure 1C left panels). Upon release from the α-factor block, cell cycle progression was inhibited and cell cycle-dependent phosphorylation of Rad9 remained severely abrogated in *cdc4-1* cells containing low CDK activity. The abrogation of Rad9 cell cycle phosphorylation is dependent upon Sic1 because cell cycle phosphorylation of Rad9 is restored in similarly treated *cdc4-1 sic1Δ* cells (indicated by retarded mobility in Figure 1C middle panels). Rad9 is phosphorylated even during α-factor arrest as a consequence of increased Cdc28/Clb activity due to the absence of Sic1 in these conditions. Rad9 phosphorylation was observed in similarly treated *cdc7-1* cells, which also block cell cycle progression and DNA replication prior to S phase but unlike *cdc4-1* cells have a low level of Sic1 and correspondingly high CDK activity (Figure 1C right panels). This confirms that Cdc28/Clb complexes are needed for Rad9 cell cycle phosphorylation. We also established that Rad9 can be phosphorylated irrespective of whether DNA synthesis takes place or not by manipulating the levels of the Cdc6 protein required for the initiation of DNA synthesis (Figure S1B). Together, our data indicate that Rad9 cell cycle phosphorylation is dependent upon B-type cyclin forms of the major CDK of budding yeast and is independent on the generation of S phase structures.

### CDK sites 1–9 in the CAD region of Rad9 are phosphorylated by Cdc28/Clb2

**Figure 2:**
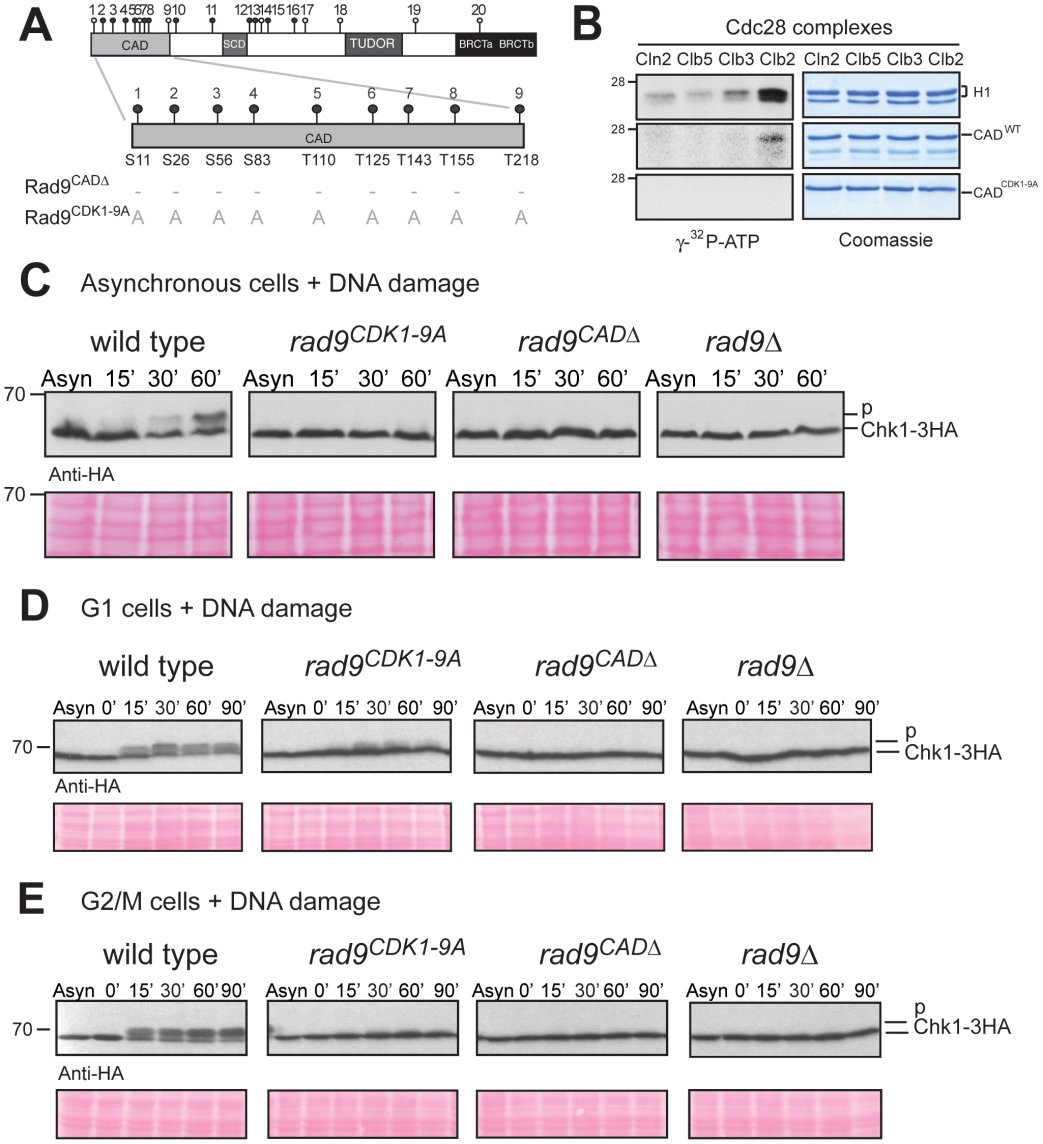
N-terminal CDK consensus sites are phosphorylated by Cdc28/Clb2 and are required for activation of Chk1 *in vivo*. (A) Schematic representation of the Rad9 N-terminus showing the 9 consensus sites for phosphorylation by Cdc28 and those mutated to alanine in Rad9^CDK1-9A^ mutant protein. (B) *In vitro* phosphorylation of Rad9 CAD^WT^, but not CAD^CDK1-9A^, requires Cdc28/Clb2. *In vitro* kinase assays were performed on the indicated substrates with four specific purified Cdc28 complexes. (C) DNA damage-dependent Chk1 phosphorylation is defective in *rad9^CDK1-9A^, rad9^CADΔ^* and *rad9 Δ* cells. Asynchronously growing cells were treated with bleocin for the indicated times and Chk1 phosphorylation analysed by western blotting. (D&E) Chk1 activation is mostly dependent on the 9 CAD CDK consensus sites in G2/M cells. Cells were grown and arrested in the cell cycle as indicated with either α-factor (G1 cells in D) or nocodazole (G2/M cells in E), treated with bleocin for the indicated times and analysed by western blotting of Chk1-3HA (D & E). See also Figure S2 for related experiments.

Nine of the 20 consensus CDK phosphorylation sites of Rad9 are located in the N-terminal 231 residues (Figure 2A) referred to as the Chk1 Activating Domain (CAD) [35] and are likely targets for Cdc28/Clb complexes. First, we established that Cdc28/Clb complexes are capable of phosphorylating the CAD region *in vitro* using the four major classes of cyclin-Cdc28 complexes as described by Kõivomägi and collaborators [55]. Essentially, the Cln2 form is G1-specific, the Clb5 form is S-specific, the Clb3 form is G2-specific and the Clb2 form is mitosis-specific. We purified these four complexes (Figure S2A) and tested their ability to phosphorylate a recombinant Rad9 CAD region relative to the standard histone H1 substrate used in Cdc28 kinase assays (Figure 2B). We observed that the Rad9 CAD region was preferentially phosphorylated by the Cdc28/Clb2 kinase with a profile similar to histone H1, although H1 was phosphorylated to higher levels (Figure 2B). Strikingly, no phosphorylation was observed when the recombinant CAD^CDK1-9A^ lacking all 9 CDK sites due to alanine substitution of the serine and threonine residues was used as a substrate (Figure 2B) even in the presence of increased amounts of kinase (Figure S2B). These results indicate that the sites targeted by the Cdc28/Clb2 complex *in vitro* are among the 9 CDK consensus sites in the CAD region.

To determine whether the 9 CDK consensus sites in Rad9 are phosphorylated *in vivo*, we then compared a mutant strain *rad9^CDK1-9A^* expressing a Rad9 protein, in which the four serines and the five threonines corresponding to CDK sites 1–9 were mutated to alanines (Figure 2A and Figure S2C), with *rad9^CADΔ^* cells, which express a truncated Rad9 protein lacking the CAD region and are defective for Chk1 activation [35], [56]. We used Western analysis to determine the extent of Rad9 cell cycle phosphorylation in *rad9^CDK1-9A^* cells (Figure S2D). The detection of slower migrating forms of Rad9^CDK1-9A^ is in agreement with our *in vitro* phosphorylation assay (see Figure 2B) and consistent with at least some of the CDK sites mutated in Rad9^CDK1-9A^ being phosphorylated *in vivo* (Figure S2D).

### CDK sites in the Rad9 CAD are specifically required for Chk1 activation in G2/M phase

The CAD region has been identified as necessary for Chk1 activation by damage-dependent phosphorylation through an unknown mechanism [35]. We hypothesized that Cdc28-dependent phosphorylation of some or all of the 9 CDK consensus sites in the CAD region (here termed CDK1-9) is important for Chk1 activation. The ability of the CDK1-9 sites to mediate Chk1 activation was assessed by analysing the DNA damage-induced phospho-shift of tagged Chk1-3HA by immunoblotting as previously described [57]. A lower mobility Chk1 phospho-form rapidly appears in response to bleocin treatment of asynchronously growing wild type cells (Figure 2C). As reported earlier, Chk1 activation in this assay was defective in *rad9Δ* and *rad9^CADΔ^* cells [35], [57]. The damage-induced Chk1 phospho-shift was also abrogated in *rad9^CDK1-9A^* cells. Similar results were obtained in response to 4-NQO (Figure S2E).

A comparison of G1 and G2/M arrested cells revealed that mutation of CDK 1–9 sites to alanine caused severe defects in Chk1 activation after bleocin treatment in G2/M cells, although there was some residual activation in G1 arrested cells (Figure 2D and 2E). Similar results were obtained with IR, 4-NQO and UV (Figure S2F). Thus, the dependence of Chk1 activation on CDK sites 1–9 correlates well with elevated CDK activity and Rad9 cell cycle phosphorylation in G2/M. Our results are consistent with a role for the CDK sites of the Rad9 CAD region in the regulation of DNA damage-induced Chk1 kinase activity.

**Figure 3:**
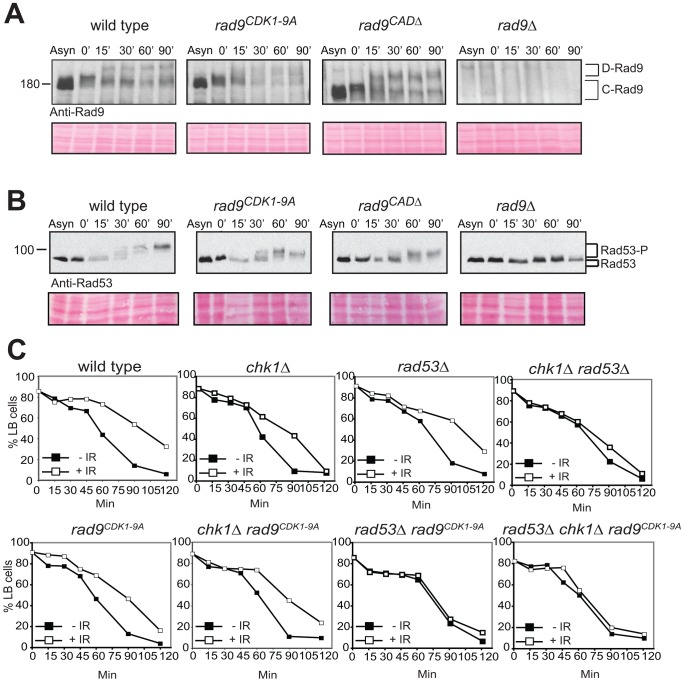
The CDK1-9 sites of Rad9 specifically function in the Chk1 branch of the G2/M checkpoint. *rad9^CDK1-9A^* mutant cells arrested in G2/M by nocodazole treatment are neither defective for Rad9 (A) nor for Rad53 (B) phosphorylations induced by bleocin treatment started at time 0. (C) The CDK1-9 sites of Rad9 function specifically in the Chk1 branch and not the Rad53 branch of the G2/M checkpoint. The indicated strains were examined for epistatic relationships using the G2/M checkpoint assay, in which cells synchronized in G2/M using nocodazole are released after irradiation into medium without nocodazole, but containing α-factor, preventing cells that have successfully completed mitosis from cycling further by arresting them in G1. This assay measures the delay in completing mitosis under DNA damaging conditions by comparing the behavior of cells that have been irradiated with IR (+IR) or not (−IR). All strains contained the *sml1Δ* mutation necessary for the viability of *rad53Δ* cells. See also Figure S3.

An important aspect of the role of the Rad9 CAD region is its specificity in Chk1 regulation [35]. We observed that the formation of D-Rad9 is largely unaffected in Rad9^CDK1-9A^ cells (Figure 3A and Figure S3A) similar to *rad9^CADΔ^* cells. We also found that activation of Rad53 in G2/M arrested *rad9^CDK1-9A^* cells after DNA damage is mostly normal (Figure 3B and Figure S3B). The minor defect in Rad53 activation we observed in both *rad9^CDK1-9A^* and *rad9^CADΔ^* cells can be explained by loss of CDK site 1 (Serine 11), known to be involved in the recruitment of Dpb11 [58], which in turn is required for efficient Rad53 activation [59], [60]. However, mutation of CDK site 1 alone does not abolish DNA damage phosphorylation of Chk1 (see below and Figure S6B) suggesting that one or more of the CDK1-9 sites are required for Chk1 activation.

To confirm that the CDK1-9 sites function principally in the Chk1 activation pathway we performed an epistasis analysis using a standard G2/M cell cycle checkpoint assay ([11] and Text S1). Cells were synchronized in G2/M using nocodazole and released through mitosis using medium without nocodazole but containing α-factor to prevent these cells from cycling further by arresting them in G1 phase. The G2/M delay measured in this assay is the DNA damage-induced delay in completing mitosis observed in treated cells compared to untreated cells. This delay requires Rad9-dependent regulation of two additive pathways involving both the Chk1 and Rad53/Chk2 checkpoint kinases [57], [61]. *rad9^CDK1-9A^* cells displayed a partially defective G2/M checkpoint similar to single deletion of *chk1Δ* or *rad53Δ* (Figure 3C). A similar partial defect was also observed in *rad9^CDK1-9A^ chk1Δ* cells, whereas *rad9^CDK1-9A^ rad53Δ* cells were completely defective in this G2/M checkpoint assay. The epistatic relationship between *rad9^CDK1-9A^* and *chk1Δ* and the additive relationship between *rad9^CDK1-9A^* and *rad53Δ* together strongly indicate that the CDK1-9 sites of Rad9 function specifically to regulate Chk1 activation in response to DNA damage.

### CDK-dependent activation of Chk1 controls Rad9/Chk1 interaction

**Figure 4:**
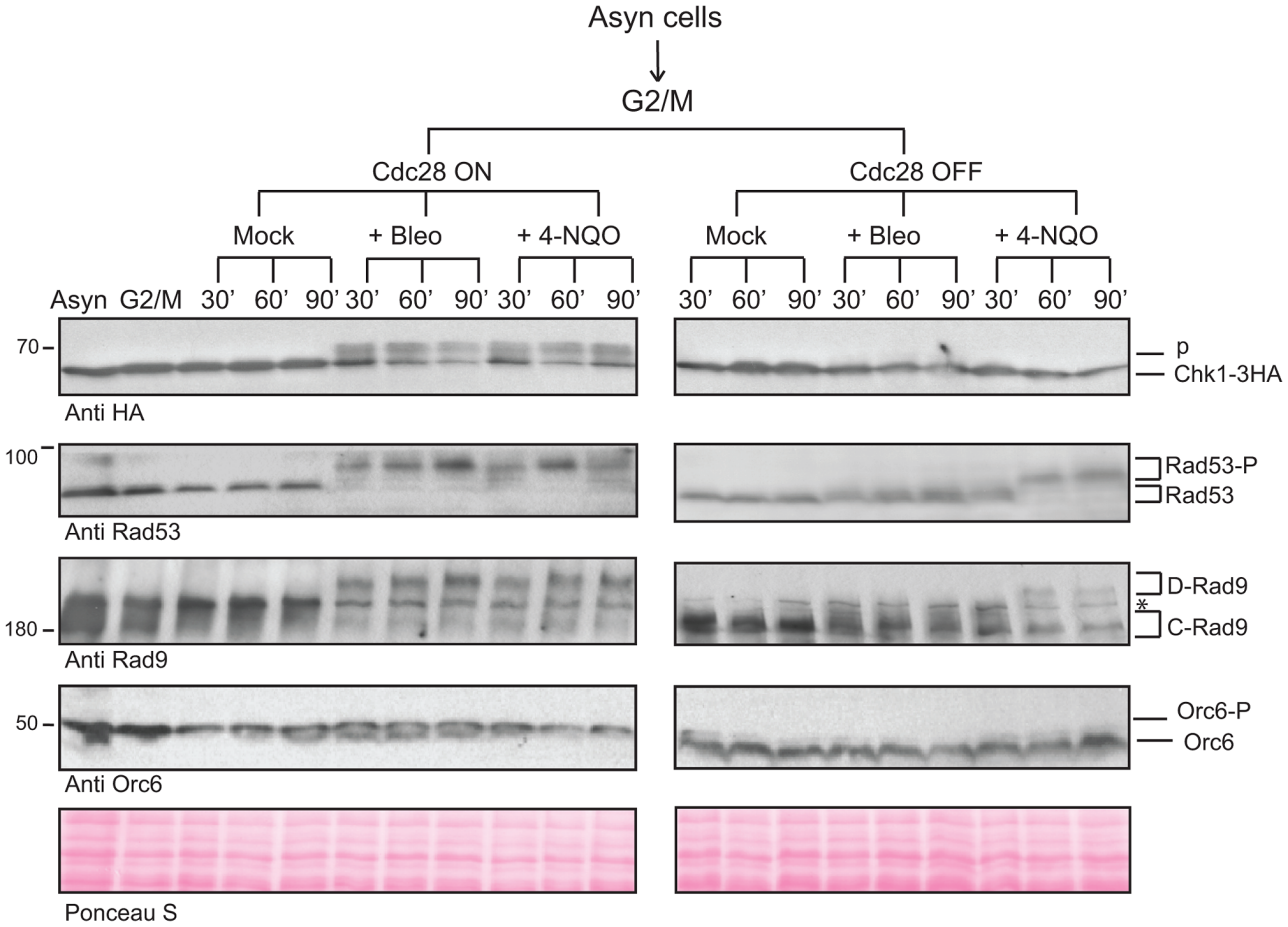
CDK is required for the activation of Chk1-dependent signaling. Cdc28 activity was regulated using the 1-NMPP1 inhibitor in G2/M arrested *cdc28-as1* cells treated with bleocin or 4-NQO to examine the activation of Chk1 signaling. Rad9 and Rad53 were followed as markers of checkpoint activation, while Orc6 phosphorylation serves as a marker for Cdc28 inactivation. See also Figure S4.

Chk1 activation in response to DSBs requires the activity of Cdc28 and this has been attributed to a key role for Cdc28 in controlling DSB resection [49], [62]. To obtain evidence for a role of Cdc28 in Chk1 control independently of Cdc28-dependent regulation of DSB resection, we used *cdc28-as1* cells, in which Cdc28 can be rapidly inhibited, arrested in G2/M and exposed to 4-NQO. These conditions do not result in DSBs as 4-NQO lesions (single strand breaks and DNA adducts repaired by NER) cannot be processed or converted in DSBs by replication forks in G2/M arrested cells. As a control we used bleocin treatment, which directly induces DSBs. Cdc28 activity was essential for Chk1 activation in response to either 4-NQO or bleocin (Figure 4 and Figure S4A). Furthermore, both Rad53 and Rad9 activation after 4-NQO treatment was independent of Cdc28 activity, unlike activation of Rad53 in response to bleocin. This is in agreement with the reported role of Cdc28 in DSB processing [62]. These results suggest that Chk1 activation upon DNA damage is absolutely dependent upon Cdc28 activity, rather than a downstream consequence of Cdc28-dependent resection that occurs at DSBs. We also observed that maintenance of Chk1 signaling requires continuous Cdc28 activity [62] (Figure S4B and S4C). Our results indicate that Cdc28 activity is essential for initiation and maintenance of Chk1 activation with all DNA damaging agents tested. They are also consistent with control of Chk1 activation by Cdc28-dependent phosphorylation of CDK sites in the N-terminus of Rad9.

**Figure 5:**
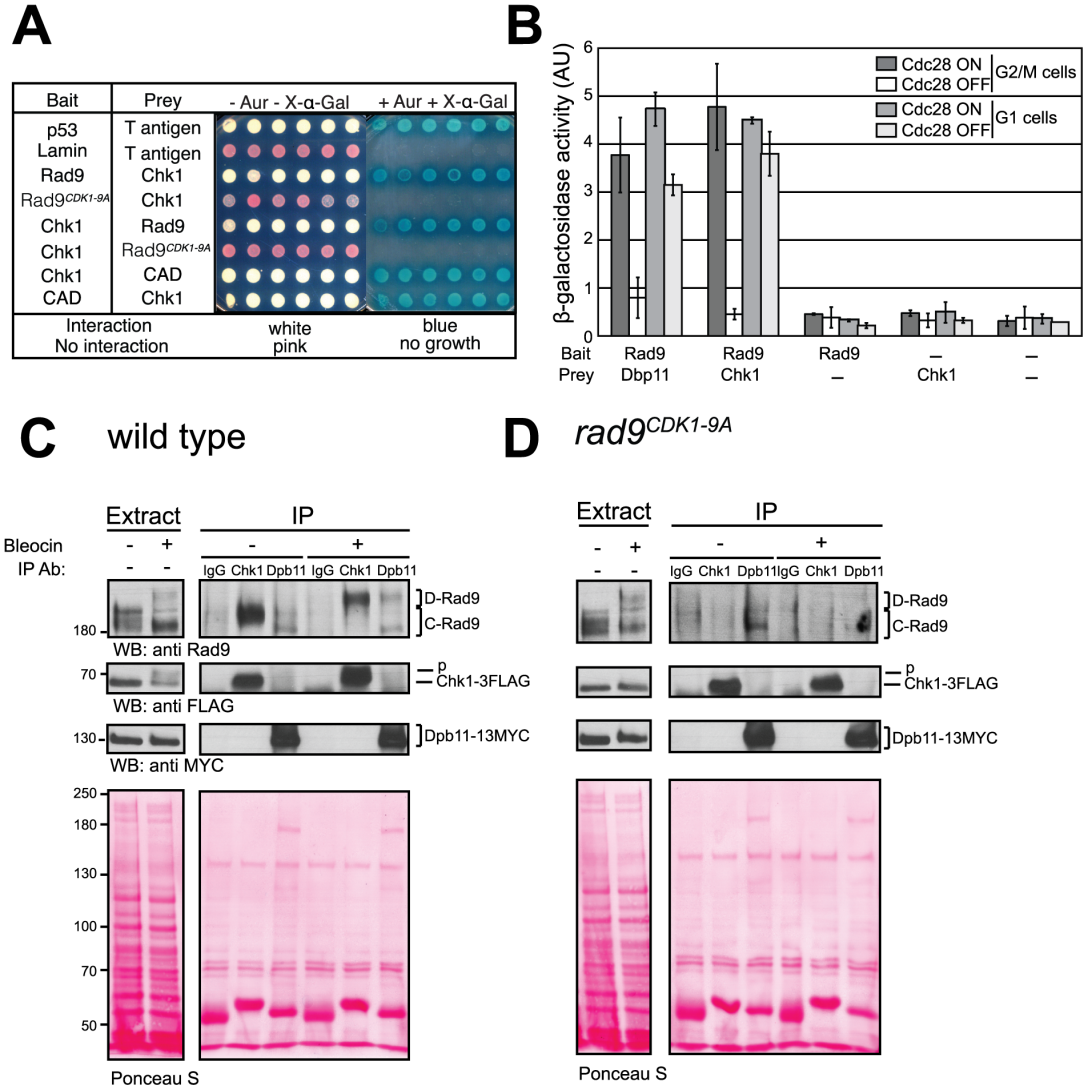
CDK-dependent phosphorylation of the nine N-terminal CDK sites in Rad9 regulates a physical interaction between Rad9 and Chk1. (A) Rad9/Chk1 interaction measured *in vivo* using a yeast two-hybrid (Y2H) assay is dependent on the CDK1-9 sites. Y2H interaction of specific bait and prey plasmids shown on the left is indicated by the white colour of the otherwise red cells, their resistance to Aureobasidin A and their blue colour on media containing the X-α-gal substrate, as for the p53/T antigen interaction control. Six independent clones are presented for each vector combination. (B) The Y2H interaction between Rad9 and Chk1 is dependent on CDK activity in G2/M cells. The indicated bait and prey plasmids were introduced into *cdc28-as1* cells mock treated or treated with 1-NMPP1 1 h after synchronization of cells with either nocodazole or alpha factor and prior to induction of expression of each bait protein. The recently reported CDK-dependent Rad9/Dpb11 interaction was used as a positive control. (C) The Rad9/Chk1 interaction measured using co-immunoprecipitation (co-IP) occurs both in the absence and presence of DNA damage. Chk1 (anti-FLAG) and Dpb11 (anti-MYC) immunoprecipitations (IPs) were performed as indicated on extracts prepared from nocodazole-arrested cells, expressing both Chk1-3FLAG and Dpb11-13MYC, and either mock treated or treated with 20 µg/ml of bleocin for 45 min. Mock (IgG) or Dpb11 (MYC) IPs were performed as controls. Rad9, Chk1-3FLAG and Dpb11-13MYC specific bands were detected in western blots. Lower exposures, to facilitate their visualisation, of the western blots of the starting extracts are shown to the left. (D) Chk1 interaction with Rad9 is dependent on the CDK1-9 sites. As in panel C, except *rad9^CDK1-9A^* cells were used. See also Figure S5 for related experiments.

A mechanistic clue as to how Chk1 could be activated by Rad9 comes from the reported interaction between these proteins in yeast two-hybrid (Y2H) assays [57]. Therefore, we tested whether this interaction depends on the 9 CDK sites in the Rad9 N-terminal region. Both full length Rad9 and the N-terminal Rad9 CAD region interact with Chk1 (Figure 5A). Importantly, the interaction between full length Rad9 and Chk1 absolutely required intact CDK1-9 sites, consistent with CDK-dependent phosphorylation of these sites regulating the interaction between Rad9 and Chk1. Using a triple-plasmid based Y2H assay (see Text S1 and [58]) we could monitor both the cell cycle and the Cdc28 dependency of the interaction by synchronising *cdc28-as1* cells in G1 and G2/M phases. In support of our hypothesis, we observed that inhibition of Cdc28-as1 activity in G2/M arrested cells resulted in loss of this interaction (Figure 5B). Consistent with the Rad9-dependent Chk1 activation observed after DNA damage observed in G1 cells (Figure 2D), the Y2H interaction between Rad9 and Chk1 was also detected in G1 arrested cells although it was clearly Cdc28-independent (Figure 5B). Therefore, a Cdc28-independent mechanism must exist that regulates the Rad9/Chk1 interaction in G1 phase. This interaction still requires the integrity of the CDK1-9 sites in G1 phase (Figure S5A) and is consistent with the decreased Chk1 activation observed in G1 arrested *rad9^CDK1-9A^* cells (Figure 2D). Importantly, our yeast two-hybrid analyses demonstrate that in the G2/M phase of the cell cycle the interaction between Rad9 and Chk1 is fully dependent on the Rad9 CAD, on the nine CDK sites located in this region, and on the activity of Cdc28 during the G2/M phase of the cell cycle.

Next, we used co-immunoprecipitation in cell extracts to study the interaction between Rad9 and Chk1 expressed at endogenous levels to confirm our Y2H Rad9/Chk1 interaction results (Figure 5C and 5D). We observed a Rad9/Chk1 interaction in immunoprecipitation experiments performed with extracts prepared from G2/M arrested wild type cells that had been either mock or bleocin-treated (Figure 5C). Rad9 can be detected in Chk1-3FLAG immunoprecipitates independently of DNA damage (Figure 5C). Notably, after DNA damage Chk1 preferentially associated with Mec1/Tel1-phosphorylated D-Rad9 even though the Cdc28-dependent C-Rad9 phospho-forms were the major forms detected in the whole cell extracts. The Rad9/Chk1 interaction was also observed in reciprocal co-immunoprecipitation experiments in which Rad9-9MYC was immunoprecipitated from G2/M arrested cells treated with bleocin (Figure S5B). Although both immunoprecipitations are efficient they do not result in significant depletion of the co-immunoprecipitated protein indicating that only a small fraction of the total pool of Rad9 and Chk1 are bound to each other in our extracts (Figure S5C and S5D). In parallel experiments we observed that the Chk1/Rad9 interaction is abolished when the CDK1-9 sites are mutated since Rad9^CDK1-9A^ is not detected in Chk1 immunoprecipitates from extracts prepared from either mock or bleocin treated *rad9^CDK1-9A^* cells (Figure 5D). Thus, co-immunoprecipitation experiments agree with our Y2H analyses and demonstrate that Rad9/Chk1 complex formation is fully dependent on the nine CDK sites located in the Rad9 CAD region. In addition, the co-immunoprecipitation experiments reveal that the Rad9/Chk1 interaction occurs independently of DNA damage.

To understand better the regulation of Chk1 activation, we compared the Rad9/Dpb11 interaction reported earlier [58] to the Rad9/Chk1 interaction reported here. After DNA damage Chk1 only interacts with D-Rad9 whereas Dpb11 can still interact with C-Rad9 as well as D-Rad9 (Figure 5C). The DNA damage interaction between Rad9 and Dpb11 was lost in bleocin treated *rad9^CDK1-9A^* cells, which is in agreement with our previous finding that Ser11 (CDK site 1) is necessary for Rad9 interaction with Dpb11 [58]. Thus, the Ser11-dependent Rad9/Dpb11 interaction is only detected after DNA damage in contrast to the constitutive CDK site-dependent Rad9/Chk1 interaction. Perhaps reflecting different abundances of Dpb11 and Chk1 in cells, the extent of Dpb11 interaction with Rad9 in the presence or in absence of DNA damage appeared much less than we observed for the Chk1/Rad9 interaction under similar conditions. Importantly, Dpb11 was never observed in the Chk1 immunoprecipitates and Chk1 was absent from Dpb11 immunoprecipitates, indicating the presence of two mutually exclusive Rad9 sub-complexes. Taken together, our results indicate that CDK-dependent phosphorylation of consensus CDK sites in the N-terminal region of Rad9 is required for an interaction between Chk1 and Rad9 that occurs both in the presence and in the absence of DNA damage treatments.

To understand how Chk1 can be released from the Rad9/Chk1 complex in response to DNA damage, we used the *in vitro* assay previously reported that demonstrated that Rad53 could be released from the Rad9/Rad53 complex *in vitro* by addition of ATP [11], [25], [26]. Rad9 complexes were washed and incubated with either ATP, its non-hydrolysable analogue ATP-γS, or mock treated. As previously reported, Rad53 became further auto-phosphorylated and was released into the supernatant in a manner dependent upon ATP hydrolysis (Figure S5E) [26]. In contrast, Chk1 phosphorylation was not further modified by ATP addition and it was not released from the Rad9 beads. These *in vitro* observations are consistent with a distinct molecular mechanism involving an ATP-dependent release from the Rad9 complex for the regulation of Rad53, but not for Chk1.

### Differential regulation of Rad9/Chk1 interaction by T125 and T143 phosphorylation

**Figure 6:**
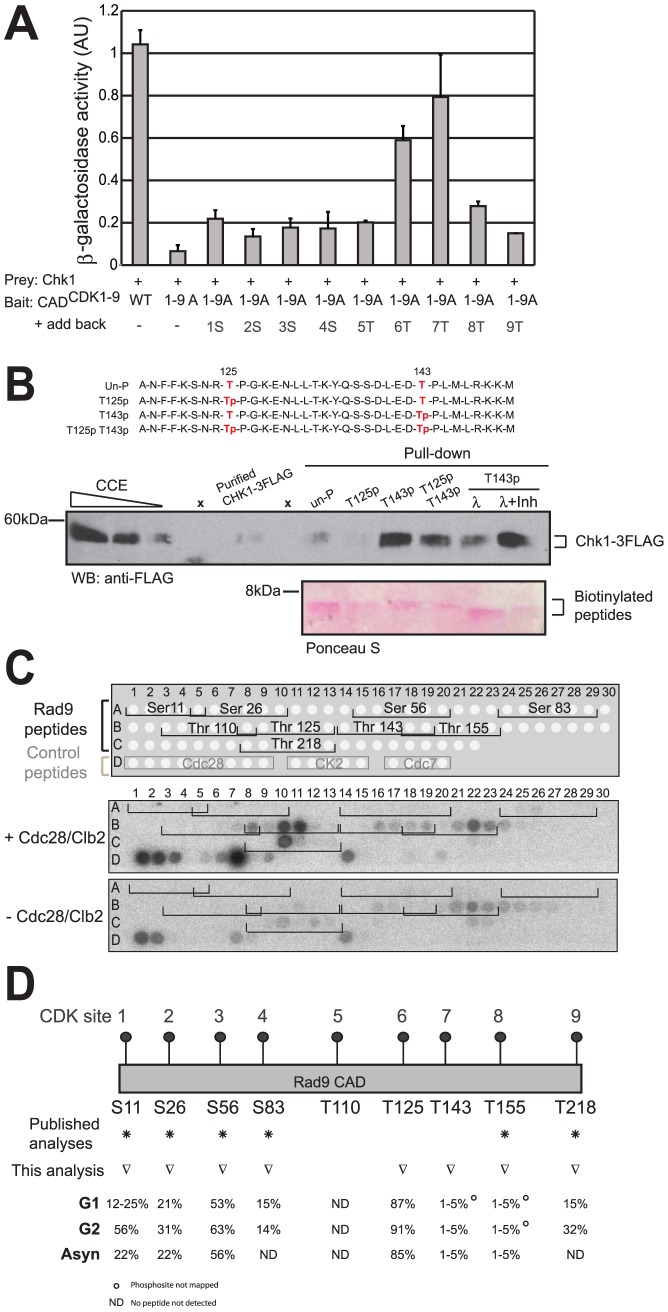
CDK sites 6 (T125) and 7 (T143) regulate the Rad9/Chk1 interaction. (A) The Y2H interaction between Rad9 and Chk1 mostly requires the 6^th^ and 7^th^ CDK sites, T125 and T143, of the CAD region (B) Peptides pull down experiments identified phosphorylated T143 (CDK site 7) and unphosphorylated T125 (CDK site 6) as the best combination of these sites required for maximum Chk1/Rad9 interaction. Chk1-3FLAG was immunopurified from *rad9Δ* cell clarified crude extracts (CCE) and incubated with four different biotinylated 35 amino-acid peptides. As represented in red, the T125 T143, T125p, T143p and T125p143p peptides were un-, mono- or di-phosphorylated on residues T125 and T143. Magnetic streptavidin beads were boiled to analyse both the presence of Chk1-3FLAG (anti-FLAG western blot) and that equal amount of peptides were used in each pull down (see Ponceau S stain). The dependency on the phosphorylation of T143 was confirmed by lambda phosphatase treatment in the absence (λ) or in the presence of phosphatase inhibitors (λ+Inh). Empty lanes are indicated by ‘x’ above the relevant lanes. (C) The Rad9 CAD peptide array phosphorylation profile shows that peptides containing consensus CDK sites 6 (T125), 7 (T143), 8 (T155) and 9 (T218) are phosphorylated by Cdc28/Clb2 *in vitro*. Peptides arrays of immobilized overlapping 19-mer peptides, each shifted to the right by 3 amino acids encompassing the first 260 amino acids of Rad9 sequence, were generated and are schematically represented at the top of the panel. The arrays were used in a kinase assay with (+) or without (−) the purified Cdc28/Clb2 complex. Target peptides of Cdc28, CK2 and Cdc7 were also spotted as controls as indicated. (D) Phosphorylation levels for CDK site-containing peptides within Rad9 isolated from asynchronous, G1- and G2/M arrested cells. Relative abundances were determined by mass spectrometry (see Material and Methods section). Residues T125 and T143 (CDK sites 6 and 7 respectively) are differentially phosphorylated *in vivo*. ○ indicates phosphorylated peptide containing this CDK site is assigned by HPLC retention time and accurate mass only. ND = Not detected. Non-phosphorylated and phosphorylated peptides were not detected. See also Figure S7, Table S1 and Table S5.

The next step to understanding the regulation of the Rad9/Chk1 interaction was to identify the specific sites in the CAD region involved. Our initial attempt to address this question using Rad9^CDK^ mutants containing different combinations of mutant sites was inconclusive (Figure S6). We therefore generated Y2H CAD mutant constructs in which each of the individual nine sites in the CAD^CDK1-9A^ mutant was mutated back to the wild type residue. The ability of these nine ‘reversion’ mutants to interact with Chk1 was assessed by the Y2H assay (Figure 6A). When alanine residues at CDK sites 6 and 7 at position 125 and 143 were individually restored to threonines, each reversion sustained significant Rad9/Chk1 interaction. The combined reversion of T125 with T143 did not further increase the Rad9 CAD/Chk1 interaction (Figure S6C). We further assessed the contribution of both positions by mutating T125 and T143 simultaneously to alanine (CAD^6A7A^) in an otherwise wild type Rad9 CAD. The Y2H interaction with Chk1 was strongly reduced, although residual interaction relative to mutation of all 9 CDK sites was detected (Figure S6D), suggesting that other sites within the Rad9 CAD might also contribute to the Rad9/Chk1 interaction. Taken together, the Y2H results reveal that the integrity of residues T125 and T143 is particularly important for the interaction between the Rad9 CAD region and Chk1.

To address whether the interaction involving residues T125 and T143 might require phosphorylation of these residues we studied the Y2H interaction between Chk1 and aspartate mutants at positions 125 and 143 in CAD^CDK1-9A^. The negatively charged aspartate residue is often used to mimic a phosphate. Substitution of aspartate at residues 125 and 143 in CAD^CDK1-9A^ resulted in interactions similar to those observed with threonines at these positions, perhaps suggesting that these residues are phosphorylated (Figure S6E). We attempted to address this issue further by measuring the Cdc28 dependency of the Y2H interaction using the phospho-mimetic mutants, but these were unstable in the absence of Cdc28 activity (Figure S6F).

As a direct and specific demonstration of the requirement for phosphorylation of residues T125 and T143 in the Rad9/Chk1 interaction, we used 35 amino-acid peptides containing unmodified, mono- or di-phosphorylated combinations of the residues to pull down Chk1 (Figure 6B). Due to the low level of Chk1 that interacts with Rad9 in extracts (Figure S5C and S5D), we used affinity-purified Chk1-3FLAG from G2/M-arrested *rad9Δ* cells. Peptides that contained phosphorylated T143 pulled Chk1 down more efficiently than peptides lacking phosphorylation at this site (compare T143p and T125pT143p with un-P and T125p in Figure 6B). Lambda phosphatase treatment of the peptide containing phosphorylated T143 reduced Chk1 interaction to the level observed for the unphosphorylated peptide (compare T143p, λ and un-P in Figure 6B), which did not occur when lambda phosphatase was inhibited (compare λ and λ+Inh in Figure 6B), confirming the importance of T143 phosphorylation for the interaction with Chk1.

Interestingly, we observed that peptides containing phosphorylation of T125 displayed reduced Chk1 interaction, independently of the phosphorylation status of T143, compared to peptides containing unphosphorylated T125. This suggests that phosphorylation of T125 has an inhibitory effect on the Rad9/Chk1 interaction. We also noted that the stimulatory effect on the interaction with Chk1 when T143 was phosphorylated was of greater magnitude on the interaction than the inhibitory effect of T125 phosphorylation. Taken together, the peptide pull-down experiments suggest that phosphorylation of T143 is required for efficient interaction with Chk1, while phosphorylation of T125 is inhibitory for this interaction.

There is an apparent discrepancy between the Y2H analyses and the peptide pull down experiments, as the former suggest that the phospho-mimetic mutation at residue 125 allows interaction with Chk1, while the latter indicates that phosphorylation of T125 is inhibitory. However, this discrepancy can be explained at least in part by an obvious caveat of Y2H experiments, the considerable overexpression of bait and prey proteins. Furthermore, Y2H experiments cannot easily address the specific requirement of phosphates at T125 and T143 because only a subpopulation of the overexpressed proteins may be phosphorylated and weak residual interactions independent of phosphorylation may be enhanced by overexpression. Moreover, phospho-mimetic mutations do not always mimic the effects of phosphorylated residues and can also be subject to overexpression artefacts.

The peptide pull down experiments suggested that phosphorylation of both T125 and T143 are key to controlling the interaction between Rad9 and Chk1, therefore we investigated whether these residues are phosphorylated *in vitro* and *in vivo*. We first established whether Cdc28 phosphorylates Rad9 residues T125 and T143 *in vitro*. To do this we used the purified Cdc28/Clb2 complex on a peptide scanning array corresponding to the Rad9 N-terminus (Figure 6C). The array consists of a library of 83 overlapping 19 mer peptides (Table S5) scanning across Rad9 residues 1–260 spot-synthesized onto nitrocellulose membranes. Cdc28/Clb2 phosphorylated a number of control peptides belonging to known Cdc28 targets (Figure 6C, see spots D1 to D9) to a much greater extent than peptides belonging to reported targets of either CK2 (spots D10 to D14) or Cdc7 kinases (spots D16 to D19). Cdc28/Clb2 was not able to phosphorylate any of the peptides containing the first five consensus CDK sites of Rad9 in this *in vitro* assay but could phosphorylate the Rad9 peptides containing T125 and T218, as well as T143 and to a lesser extent T155. This suggests that T125 and T143 are potential targets of the Cdc28/Clb2 kinase.

To determine whether T125 and T143 can be phosphorylated *in vivo*, we purified Rad9 from asynchronously growing cells and G1 or G2/M arrested cells (Figure S7A). Samples were treated with proteases and the resulting peptides were analyzed by mass spectrometry (Table S1). Tandem mass spectra obtained by either collision-activated dissociation or electron transfer dissociation of peptides from the CAD region of Rad9 provided clear evidence for phosphorylation *in vivo* on both T125 and T143 (Figure 6D, Table S1, Figure S7B), two residues not previously reported as phosphorylation sites [45]–[47]. Phosphorylation was also detected on the known sites, S11, S26, S56, S83, S137, T155, and T218 (Figure 6D, Table S1) [45]–[47]. In G1 arrested cells, six of the consensus Rad9 CDK sites (S11, S26, S56, S83, T125 and T218) were found to be phosphorylated. Increased levels of phosphorylation were detected on several of these sites on peptides from G2/M arrested cells (Figure 6D, Table S1). Near stoichiometric levels of phosphorylation were observed on peptides containing T125 for asynchronous, G1- and G2/M arrested cells. In contrast, phosphorylation levels observed for T143 containing peptides ranged from 1–5% in all three of the above samples.

Taken together, our results are consistent with Cdc28-dependent phosphorylation of T125 and T143 in the Rad9 CAD region. Phosphorylation of T143 appears to facilitate the Rad9/Chk1 interaction whereas phosphorylation of T125 is inhibitory. The combination of low levels of a stimulatory phosphorylation at T143 and high level of an inhibitory phosphorylation at T125 would function to limit the basal pool of Rad9 that can interact with Chk1.

## Discussion

### Complex cell cycle phosphorylation of Rad9 by Cdc28/Clb complexes

We show that the *S. cerevisiae* Rad9 DNA damage response mediator undergoes a complex pattern of phosphorylation during the cell cycle. Both the cell cycle specificity of modification and genetic evidence implicate Clb1-6 B-type cyclin forms of Cdc28 in this regulation (Figure 1). The *in vivo* evidence demonstrating Cdc28/Clb-dependent phosphorylation of Rad9 by Clb1-6 forms of Cdc28 is supported by previous *in vitro* studies demonstrating phosphorylation of Rad9 by purified Cdc28/Clb5 and Cdc28/Clb2 complexes [44]. It is likely that all forms of Cdc28/Clb complexes contribute to Rad9 cell cycle phosphorylation, switching from Clb5 and 6, followed by Clb3 and 4 and finally Clb1 and 2 as cells traverse the S, G2 and M phases. *In vitro* kinase assays demonstrated that the phosphorylation of the N-terminal 250 amino acid region of Rad9 was mostly dependent on Cdc28/Clb2 complexes (Figure 2B), suggesting that this form of the kinase might also be more specific for this N-terminal region. However, although less than observed in G2/M arrested cells, significant phosphorylation of Rad9 N-terminus was also detected in extracts from G1 arrested cells (Figure 6D), which have extremely low Clb-dependent kinase activity, suggesting that other kinases also contribute to phosphorylation. Our results are inconsistent with a simple threshold model of phosphorylation for regulation of the N-terminal region. They suggest either that phosphorylation of some of these sites is irrelevant or redundant to Rad9 function or, consistent with our findings for the role of Rad9 in Chk1 activation, that specific combinations of phosphorylated or un-phosphorylated CDK sites may regulate specific functions.

DDR mediators from higher cells such as 53BP1, BRCA1 and MDC1 are also rich in consensus CDK sites and some of these have been identified as sites of phosphorylation *in vivo*. It is likely that modulation of the DDR by CDK-dependent phosphorylation of mediators is evolutionarily conserved. For example, CDK-dependent phosphorylation of S379 of mouse 53Bp1 is required for binding to the mitotic kinase Plk1 [63], and S1497 of human BRCA1 is needed for its subcellular localization [40].

### Chk1 activation is dependent on Cdc28 phosphorylation of Rad9 in G2/M cells

The first 250 amino acids of Rad9, just under a fifth of the total protein, contain half of its consensus CDK sites as well as the previously identified Chk1 Activation Domain [35]. The molecular mechanisms by which the Rad9 CAD region activates Chk1 were not previously determined. Here, we establish that the 9 CDK sites located in the Rad9 N-terminal CAD region are targets for phosphorylation by Cdc28/Clb2. We also demonstrate that these 9 sites are specifically required for Chk1 activation in response to DNA damage, but not for Rad53 activation. Consistent with a role for these CDK sites, Cdc28 is also required for Chk1 activation in response to multiple DNA damaging agents including those that cause DSBs or bulky lesions repairable by nucleotide excision repair. Unlike DSB specific activation of Rad53, Chk1 regulation cannot simply be a downstream consequence of the involvement of Cdc28 in regulating DSB specific responses such as DSBs resection [64], [65] or the reported Cdc28-dependent role for Rad9 in the inhibition of ssDNA generation at DNA ends [66].

The mechanism by which CDK site phosphorylation of the Rad9 CAD region regulates Chk1 involves a direct physical interaction between the CAD region and Chk1. Surprisingly, Y2H analyses suggest that the Rad9/Chk1 interaction detected in G1 cells also requires interaction of the CDK1-9 sites with Chk1. However, in G1 cells the Y2H interaction between Rad9 and Chk1 does not require Cdc28 activity and might even be independent of phosphorylation. Taken together, our results establish that in G2/M cells when Cdc28/Clb kinases are most active Cdc28-dependent phosphorylation of CDK sites in the CAD region of Rad9 acts to regulate the activation of Chk1 *via* its interaction with Rad9.

### Residues T125 and T143 reciprocally regulate the Chk1/Rad9 interaction

While other residues in the Rad9 CAD domain may perform regulatory roles (e.g. serine 11 implicated in the Rad9 and Dpb11 interaction [58]), our Y2H analyses point to T125 and T143 as key residues for the interaction between Rad9 and Chk1. Specifically, reintroduction of the T125 and T143 wild-type residues into the nine alanine mutant (CAD^CDK1-9A^) restored the interaction between Rad9 and Chk1. Similarly, the introduction of a negative charge at these positions using aspartate substitutions, that may mimic phosphorylation, is also permissive for the Rad9/Chk1 interaction. Conversely, a mutant where both these residues were substituted to alanine in an otherwise wild-type Rad9 sequence was defective for the Rad9/Chk1 interaction. Thus T125 and T143 are part of a Rad9/Chk1 interaction module, which can tolerate substitution to aspartate but not alanine. We directly assessed the consequence of the phosphorylation status of T125 and T143 on their ability to interact with Chk1 using peptide-pull down experiments. Interestingly, we found that phosphorylation of T143 stimulates the Rad9/Chk1 interaction, whereas phosphorylation of T125 is inhibitory to this interaction.

Importantly, we have established that these two residues are phosphorylated not just *in vitro* but also *in vivo*. While T125 is a major site of phosphorylation, T143 is only a minor site of phosphorylation. The dual regulation of the Rad9/Chk1 interaction by these residues, where the optimal configuration is phosphorylation of T143 but not T125, would ensure only a small proportion of Rad9 molecules interact with Chk1. This is consistent with our observations using immunoprecipitation where we find that most Rad9 molecules are not bound to Chk1 and can form other complexes that do not contain Chk1. Dual regulation of Rad9 by phosphorylating T143 and dephosphorylating T125 may also fine-tune Rad9/Chk1 complex formation in response to DNA damage. For example, regulated de-phosphorylation of T125 by a specific phosphatase-containing complex associated to phosphorylation of T143 could be used to enforce or maintain Chk1 signalling subsequent to the initial activating event. The ability to manipulate the Rad9/Chk1 interaction with stimulatory and inhibitory phosphorylation events provides interesting possible mechanisms for its temporal and/or spatial regulation. Taken together, our results indicate that T125 and T143 constitute important components of the interaction surface on Rad9 that binds to Chk1, in which phosphorylation of these residues contributes either negatively of positively respectively to the docking of Chk1 onto the Chk1 Activating Domain of Rad9.

### Molecular mechanism regulating Chk1 activation

**Figure 7:**
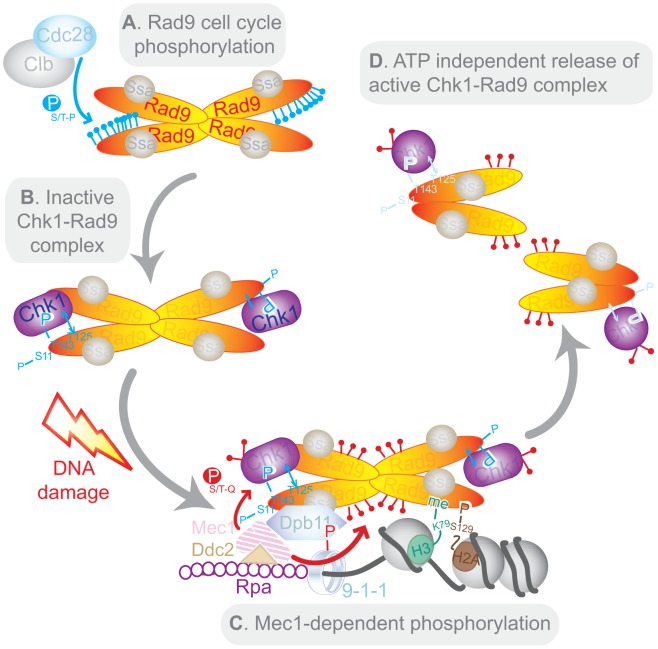
Model of Chk1 activation in response to DNA damage. Note that the tetramer representation of Rad9 is a possible conformation for the C-Rad9 oligomeric complex of greater than or equal to 850 kDa, which is remodelled into a smaller complex of approximately 560 kDa containing D-Rad9 after DNA damage. See text for a description of the model.

Our results are consistent with a novel model for Chk1 activation (Figure 7). The Rad9/Chk1 interaction is DNA damage-independent but regulated during S/G2/M by Cdc28/Clb-dependent modification of Rad9 (Figure 7A). The Rad9/Chk1 interaction is very likely to be most stimulated when T143 is phosphorylated and T125 is unphosphorylated (Figure 7B). Rad9 and Chk1 are both believed to dynamically associate with chromatin even in the absence of DNA damage [58], [67], [68]. Rad9 complexes undergo enhanced chromatin recruitment in the vicinity of damaged DNA by exposure to or generation of specific histone marks such as H3K79me2 and γH2A [67], [69]. Other sites of phosphorylation in Rad9 are involved in its recruitment to Dpb11, which is recruited in a 9-1-1 complex-dependent manner [60] (Figure 7C). The Rad9/Dpb11 interaction can be either direct *via* Rad9 T462 and T474 [70] or indirect *via* Rad9 S11 [58]. Together, the 9-1-1 and Dpb11 complexes enhance the kinase activity of Mec1 bound to RPA-coated ssDNA in a mechanism that is conserved from yeast to human cells [59]. Mec1 kinase activity at sites of DNA damage results in DNA damage-dependent phosphorylation and remodelling of both Rad9 [25] and Chk1 [29], which in turn leads to *in cis* autophosphorylation of Chk1 [31] and subsequent release of the D-Rad9/Chk1 complex from damaged chromatin (Figure 7D). The Rad9/Chk1 complex is a constitutive but minor Rad9 complex. It is recruited along with other Rad9 complexes to sites of DNA damage where Rad9 is converted from its C- to D-form, irrespective of its interaction with Chk1. Thus, after DNA damage Chk1 interacts specifically with D-Rad9. Interestingly, activation of the Chk1 kinase contrasts with Rad53 (also known as Chk2) activation which involves its *in trans* auto-phosphorylation subsequent to Mec1 (ATR)-dependent priming and its specific interaction with D-Rad9 generated in response to DNA damage [27]. Additionally, *in vitro* experiments suggest that activated Rad53 is released from Rad9 in an ATP-dependent mechanism [26] which we did not observe with Chk1. Release of Chk1 from Rad9 could be controlled by the reversal of the phosphorylation status of residues T125 and T143. Alternatively, it could also be controlled by the de-phosphorylation of PIK kinase-dependent sites of phosphorylation on Chk1 at a later stage of the response as has been reported for human Chk1 [30]. While our findings predict that de-phosphorylation of T143 and phosphorylation of T125 would destabilise the interaction between Rad9 and Chk1, the maintenance of T143 phosphorylation and lack of T125 phosphorylation could keep the Rad9/Chk1 interaction intact in the proximity of damaged chromatin where activated Chk1 could then phosphorylate local targets.

### Conservation of Chk1 activation mechanisms

Together with a recent report [71], our work constitutes the first detailed characterisation of Chk1 activation in the major yeast model systems. The fission yeast homologue of Rad9, Crb2 interacts with Chk1 through a Chk1-binding module that also involves two N-terminally located phosphorylated residues, T73 and S80 [71]. However, these residues are not conserved in Rad9 and are not sites of CDK-dependent phosphorylation. Instead phosphorylation of Crb2 residues T73 and S80 is dependent upon DNA damage and Rad3, the Mec1/ATR homologue in fission yeast [71]. Interestingly, the principal role for Crb2 and Rad9 in Chk1 activation, its recruitment to sites of DNA damage to allow its activation by PIKKs, is conserved between budding and fission yeasts. However, the two yeasts achieve this role by divergent mechanisms. Fission yeast Crb2 is using a DNA damage-recruitment mechanism stimulated by Rad3-phosphorylated docking sites. In contrast, in budding yeast the constitutive Rad9 sub-complex is found in cycling cells that have not been subject to exogenous DNA damage and both Rad9 and Chk1 are co-recruited on damaged chromatin.

It is possible that the differences between Chk1 regulation in budding and fission yeast reflect the difference in regulating the cell cycle in the two yeasts. Budding yeast has a clearly defined G1 phase, whereas fission yeast normally does not. It is also possible that the DNA damage-independent Rad9/Chk1 interaction indicates a yet unidentified role for this complex in the absence of DNA damage. Alternatively, or perhaps in addition, budding yeast regulation of the Rad9/Chk1 interaction might reflect the more discrete and specific roles of *S. cerevisiae* Chk1 kinase in checkpoint regulation and the need to limit Chk1 activation to its specific contexts. In contrast, *S. pombe* Chk1 is the main DNA damage checkpoint kinase, fulfilling roles equivalent to that of the Rad53 kinase in budding yeast, and, therefore, Rad3-dependent regulation of the Crb2/Chk1 interaction might allow a less restricted activation of the *S. pombe* kinase. Even further complexity in the regulation of Chk1 activity is suggested by the regulation of the Claspin/Chk1 interaction by the constitutively active Casein kinase 1 gamma 1 in human cells [72]. Nevertheless, Chk1 recruitment to sites of DNA damage is conserved from yeast to human cells, since recruitment of DNA damage mediators to damaged chromatin is dependent upon the Mec1/Rad3/ATR kinases [24].

In higher cells, the role played by DDR mediator proteins is more complex than in yeasts as multiple mediators are known and their molecular mechanisms in checkpoint activation remain largely uncharacterised [4], [32]. Among human mediators, Claspin-dependent activation of CHK1 is best characterized, although BRCA1 and MDC1 also play undefined roles in this activation. Our work suggests that activation of human CHK1 by mediator proteins, possibly those related to budding yeast Rad9, may also be integrated into cell cycle stage by their prior CDK-dependent phosphorylation and dynamic interaction with CHK1. Cell cycle-dependent phosphorylation may also fine-tune other functions of mediator proteins.

## Materials and Methods

### Strains

All strains used in this work are listed in Table S2 and in the W303 background (*MATa ade2-1 trp1-1 can1-100 leu2-3,12 his3-11,15 ura3*) with the exception of CG378 used in Figure 1A and strains used to purify the Cdc28 complexes. All mutant and tagged alleles used were integrated on the chromosome apart from the yeast two-hybrid experiments. Yeast strain (Table S2) and plasmid constructions (Text S1) are described in supplementary material. Plasmids and oligonucleotides used in this study are listed in Tables S3 and S4.

### Cell cycle arrest and checkpoint experiments

These experiments were performed as described earlier [11] and are described in detail in supplemental methods (Text S1).

### Antibodies

Western blotting was performed as previously described [11], [20]. Rad9 phosphoforms, Rad53, Swi6, Dpb11-13MYC, RAD9-9MYC and Chk1-3FLAG were probed with NLO5 and NLO16 [11], [20] or Abcam anti-Rad53 (ab104232), NLO2 (D. Lee & N. Lowndes), anti-MYC (9E11, Abcam) and anti-FLAG (M2, Sigma) antibodies respectively. Sic1, Clb2, Chk1-3HA, CAD-HA and Orc6 were with anti-Sic1 (JD156 from J. Diffley), anti-Clb2 (sc9071 from Santa Crutz), anti-HA (12CA5) and anti-Orc6 (SB49 from B. Stillman) antibodies respectively.

### *In vitro* kinase assay using specific Cdc28 complexes

The purification of the four Cdc28 complexes and the *in vitro* kinase assays were performed as described previously [55].

### Yeast two-hybrid

Interactions were assessed using Clontech Matchmaker TM Gold Yeast Two Hybrid System (Catalog no 630489) according to the manufacturer's instructions. A triple-plasmid based assay was used to study the Cdc28-dependent interaction between Rad9 and Chk1 proteins as described earlier [58].

### Yeast native extracts and immunoprecipitations

Clarified crude extracts (CCE) of wild type or *rad9^CDK1-9A^* cells, expressing either both Chk1-3FLAG and Rad9-9MYC or both Chk1-3FLAG and Dpb11-13MYC, arrested in G2/M phase and either mock treated or treated with 20 µg/ml of bleocin (Calbiochem) for 45 min were generated as previously described [58]. One ml of CCE at a concentration of 10 mg/ml was used in immunoprecipitation experiments as previously described [58] with anti-FLAG, anti-MYC or IgG antibodies. Beads were finally resuspended in 40 µl of 3× Laemmli buffer, boiled for 5 min and released proteins were separated on SDS-PAGE gels for western blot analysis. Note that all tagged strains were confirmed for functionality (Figure S7C).

### Peptide pull-down experiment

Chk1-3FLAG was immunopurified from *rad9Δ* cell crude extracts as described in supplementary material. Biotin-labelled Rad9 peptides, synthetised by Pepceuticals Limited (Leicestershire, UK), were used to saturate pre-washed Streptavidin Dynabeads M-280 (Invitrogen). In experiment involving phosphatase treatment, peptides beads were treated with 10 unit of λ phosphatase for 20 min at 30°C in the presence or in the absence of phosphatase inhibitors. Immunopurified Chk1-3FLAG was added to the peptides beads and incubated for 2 hours, on a rotating wheel at 4°C. The washed beads were resuspended in sample buffer, boiled at 95°C, loaded onto a 20% SDS-PAGE gel for peptide detection by Ponceau staining or on a 6.5% 80/1 acrylamide/bis-acrylamide SDS-PAGE gel to visualize Chk1-3FLAG.

### SPOT synthesis of peptides and array experiment

The peptides arrays of Rad9 on nitrocellulose were generated as previously described [73]. Essentially, scanning libraries of overlapping 19-mer peptides covering the 260 first amino-acids of Rad9 (listed in Table S5) were produced by automatic SPOT synthesis and synthesized on continuous cellulose membrane supports on Whatman 50 cellulose using Fmoc (9-fluorenylmethyloxycarbonyl) chemistry with the AutoSpot-Rosbot ASS 222 (Intavis Bioanalytical Instruments). Peptide arrays were submitted to Cdc28/Clb2 kinase assays.

### Rad9 mass spectrometry analysis

A two-step immunopurification of Rad9-GFP-FLAG was performed on crude cell extracts obtained from asynchronous, G1- and G2/M arrested cells (Text S1). Peptides from purified Rad9-GFP-FLAG protein were analyzed by collision-activated dissociation and/or electron transfer dissociation tandem mass spectrometry (Text S1). Phosphopeptides detected from the CAD region of Rad9 are presented in Table S1. Percent relative abundances for the Rad9 phosphopeptides were calculated from the ion currents observed in main beam mass spectra [(phosphorylated peptide ion current)/(phosphorylated peptide+non- phosphorylated peptide ion current)×(100)]. Phosphorylation sites were assigned by manual interpretation of collision-activated dissociation and/or electron transfer dissociation tandem mass spectra obtained for each phosphopeptide species. All phospho-peptide signals were detected at signal/noise levels greater than 100 (S/N = >100).

## Supporting Information

Figure S1 Related to Figure 1. Cell cycle phosphorylation of Rad9 is not dependent on DNA replication structures but on multiple potential CDK phosphorylation sites in Rad9. (A) Rad9 is phosphorylated in asynchronous cells in absence of DNA damage. The slow migrating forms of Rad9 caused by cell cycle phosphorylation disappear in response to λ phosphatase treatment. (B) Rad9 phosphorylation is independent from the initiation of DNA synthesis. Cell cycle phosphorylation of Rad9 was examined in a cell cycle engineered to bypass S phase, by manipulating the level of the Cdc6 replication protein. In the absence of Cdc6 cells undergo a haploid mitosis in which the monovalent chromosomes are randomly segregated to either pole. Cells in which Cdc6 was either present or absent were released from a late mitotic block (the *cdc15-2*2 mutation at 36°C) [74] and examined for Rad9 cell cycle phosphorylation and progression through the cell cycle. Rad9 was normally phosphorylated irrespective of whether DNA synthesis took place or not. Thus, although Rad9 phosphorylation during cell cycle progression is dependent upon Cdc28/Clbs complexes, it is independent of DNA structures generated during a normal S phase.
(EPS)

Figure S2 Related to Figure 2. The CDK1-9 sites within the CAD region of Rad9 are phosphorylated both *in vitro* and *in vivo*. (A) Silver-stained gel of the purified Cdc28/Cln complexes used in this study. * indicates a contaminant in the Cdc28/Cln2 purification. (B) Cdc28/Clb2 phosphorylates Rad9 CAD^WT^ but not CAD^CDK1-9A^ *in vitro*. *In vitro* kinase assays were performed on the indicated substrates with higher concentration of Cdc28/Clb2 complex than the one presented in Figure 5D (3.6 nM compared to 0.6 nM). * indicates degradation product of Rad9 CAD^WT^. (C) Cells expressing Rad9^CDK1-9A^ as their only Rad9 protein are not sensitive to the indicated DNA damaging treatments. Drop tests were performed in the indicated strains. Note that the bleocin sensitivity of proliferating *chk1Δ* cells could indicate a role for *CHK1* in surviving bleocin-induced lesions during S phase, which can be rescued by a transient arrest at the G2/M transition induced on nocodazole plates. This role is clearly independent from the N terminus of Rad9. (D) Rad9^CDK1-9A^ displays defective cell cycle and Cdc28-dependent phosphorylation *in vivo*. Rad9 western blot prepared from the indicated strains. Rad9^12A^ is the short name of a strain expressing *rad9^CDK1,3,4,6,9,11,14,16-20A^* (Karen Finn, Unpublished data). 1-NMPP1 treatment of *cdc28-as1* cells was used to indicate Cdc28-dependent phosphorylation. (E) DNA damage-induced Chk1 phosphorylation is defective in *rad9^CDK1-9A^, rad9^CADΔ^* and *rad9 Δ* cells. Asynchronously growing cells were treated with 4-NQO for the indicated times and Chk1 phosphorylation analysed by western blotting. (F) IR, 4-NQO or UV-induced Chk1 phosphorylation is abolished in nocodazole arrested *rad9^CDK1-9A^*, *rad9^CAD^* and *rad9Δ* cells, but there is residual Chk1 activation partially dependent on the CDK1-9 sites in G1-arrested cells.
(TIF)

Figure S3 Related to Figure 3. The CDK1-9 sites within the CAD region of Rad9 are not required for damage-induced Rad9 and Rad53 phosphorylations. (A) Rad9 DNA damage-induced phosphorylation is not dependent on the CDK1-9 sites in G2/M-arrested cells after IR and in asynchronously growing cells after 4-NQO. (B) Rad53 DNA damage-induced phosphorylation is not dependent on the CDK1-9 sites in G2/M-arrested cells after IR or 4-NQO and in asynchronous cells after 4-NQO.
(TIF)

Figure S4 Related to Figure 4. Cdc28 activity is required for initiation and maintenance of DNA damage-induced Chk1 activation in G2/M cells. (A) CDK-dependency of the initial IR (+400 Gy) induced phosphorylation of Chk1. Cdc28 was inactivated with 1-NMPP-1 in half of the G2 arrested cells and treated with IR to initiate the checkpoint in populations with and without Cdc28 activity. Protein samples were collected at the indicated time points and Chk1 phosphorylation analysis was performed. Orc6 phosphorylation is used as a control for Cdc28 inactivation in all experiments. (B) CDK-dependency of the maintenance of bleocin- induced Chk1 phosphorylation. 1-NMPP1 was added into half of the G2/M arrested and bleocin-treated cells to inactivate Cdc28 activity. Chk1 phosphorylation analysis was performed from protein extracts collected at the indicated time points. Cdc28 activity was regulated using the 1-NMPP1 inhibitor in G2/M arrested *cdc28-as1* cells treated with bleocin or 4-NQO to examine the maintenance of Chk1 signaling. Rad9 and Rad53 were followed as markers of checkpoint activation. (C) CDK-dependency of the maintenance of IR (+400 Gy) induced Chk1 phosphorylation. 1-NMPP1 was added into half of the G2/M arrested and IR-treated cells to inactivate Cdc28 activity. Chk1 phosphorylation analysis was performed from protein extracts collected at the indicated time points.
(EPS)

Figure S5 Related to Figure 5. Interaction between Rad9 and Chk1 is dependent on the Rad9 CDK1-9 sites. (A) The Y2H interaction between Rad9 and Chk1 is dependent on the CDK1-9 sites in both G1 and G2/M cells. The indicated bait and prey plasmids introduced into Y2H cells (identical to clones shown in S5A) were grown, divided into two flasks and arrested in G2/M and G1 phases of cell cycle. 1 ml of cells corresponding to one OD value were used to perform the PNP assay (see supplementary information). The α-galactosidase activity was measured according to Clontech Yeast Two Hybrid instructions. (B) Western blotting analysis of the indicated proteins in a reciprocal IP using Rad9-9MYC and Chk1-3FLAG expressing cells confirms the Rad9 and Chk1 interaction. Anti-MYC antibodies were used with extracts from nocodazole-arrested cells, treated with 20 µg/ml of bleocin for 45 min and a mock (IgG) control was performed. Rad9 binding to Rad53 was used as a further control. Different exposures of the crude extracts and the IPs lanes are shown to allow visualization of Rad9-9MYC, Chk1-3FLAG and Rad53 specific bands. (C) Western blotting analysis of the supernatants resulting from the IP experiment using Chk1-3FLAG and Dpb11-13MYC expressing cells presented in Figure 5C. (D) Western botting analysis of the supernatants resulting from IP experiment using Rad9-9MYC and Chk1-3FLAG expressing cells presented in Figure S5B. (E) ATP-dependent release of Rad53, but not Chk1 from Rad9 IPs. Assays were performed as described (Gilbert et al, 2001) except Rad9-9MYC was immunoprecipitated using an anti-MYC monoclonal antibody. The extract was prepared from nocodazole-arrested cells, treated with 20 µg/ml of bleocin for 45 min that expressed both Rad9-9MYC and Chk1-3FLAG. The amount of Rad9-9MYC, Chk1-3FLAG or Rad53 remaining on the beads (Beads) or released (Elution) after incubation with ATP (+), ATP-γS (γS), a non-hydrolysable analogue, or mock treatment without any nucleotide (−) was determined by western blotting. Different exposures of the crude extracts, elutions and beads lanes are shown to allow visualization of Rad9, Chk1 and Rad53 specific bands. • indicates the signal detected from the IgG heavy chains.
(TIF)

Figure S6 Related to Figure 6. T125 and T143 are important for Rad9/Chk1 interaction. (A) Schematic representation of the different combinations of mutated *CDK* sites in the CAD region of Rad9. (B) IR-induced Chk1 activation analysis in *rad9^CDK^* intermediate mutants shown in (A) covering all the sites in the CAD region. The single consensus PIK site (T16/A) in the CAD region was also tested (in *rad9^CDK1,2,PIK1A^* mutant) for IR-induced Chk1 phosphorylation, but had no effect. G2/M arrested wild type and mutants were treated with IR and cells were collected after 30 minutes for budding index and western blot analysis. (C) The Y2H interaction between Rad9 CAD and Chk1 is restored by the reversion to a wild-type residue of the alanine mutation of the 6^th^ and 7^th^ CDK sites (T125 and T143 respectively) in the CAD^CDK1-9A^ mutant. The analysis of the CAD^CDK1-9A+6T^, CAD^CDK1-9A+7T^ and CAD^CDK1-9A+6T7T^ (labelled 6T, 7T and 6T7T) shows that the two residues do not contribute additively to this Y2H interaction. (D) The Y2H interaction between Rad9 CAD and Chk1 is affected when both the 6^th^ and 7^th^ CDK sites, T125 and T143 respectively, are mutated to alanine a non-phosphorylatable residue. Note that all the other consensus sites are wild type in the 6A7A (CAD^CDK6A+7A^) mutant. (E) The Y2H interaction between Rad9 CAD and Chk1 is restored by the reversion of the alanine mutation of both the 6^th^ and 7^th^ CDK sites, T125 and T143 respectively, to aspartate, a phosphomimetic residue in the CAD^CDK1-9A^mutant. The mutants CAD^CDK1-9A+6D^, CAD^CDK1-9A+7D^ and CAD^CDK1-9A+6D7D^ are labelled 6D, 7D and 6D7D. Note that the 7D and 6D7D mutants restore the interaction to the same extent as the 6T7T (CAD^CDK1-9A+6T7^) wild type residue reversion. (F) The Rad9 CAD region overexpressed in the Y2H triple plasmid based assay is unstable when Cdc28 is inactivated. Interaction between Rad9 CAD, CAD^CDK1-9A^, CAD^CDK1-9A+6T^ or CAD^CDK1-9A+7T^ (labelled WT, 1-9A, 6T and 7T respectively) and Chk1 has been quantified in *cdc28-as1* cells arrested in G2/M and treated with NMPP1 (Cdc28 −) or not (Cdc28 +). Over-production of the CAD regions was detected by Western blot analysis and is shown in the bottom panel.
(EPS)

Figure S7 *In vivo* analysis of Rad9 Chk1 Activation Domain phosphorylation status. (A) Silver stained gel of the purified Rad9-GFP-FLAG used in ETD-Mass spectrometry analyses shown in Figure 6D and Table S1. This gel shows the result of the second immunopurification step (GFP) described in supplemental methods. The Flag elution (Load) was incubated with GFP-Trap beads. The Rad9-GFP-FLAG retained on these beads (Beads) was used for the analyses. The unbound fraction (FT) shows the presence of two unspecific high molecular weight bands not detected in Rad9 western blotting. (B) Extracted Ion Chromatograms (XIC) and Main Beam Mass Spectra (MS) obtained for the T143-containing Rad9 peptides isolated from G2-arrested cells (YQSSDLEDTPLMLRK – Top Panel and YQSSDLEDpTPLMLRK – Bottom Panel). Signal to noise levels for this phosphopeptide and all others in Table S1 are >100 (S/N = >100). (C) Drop test analysis of the indicated tagged strains used for immunoprecipitation experiments shown in Figure 5 and Rad9 purification used for mass spectrometry analyses in Figure 6D, Table S1 and Figure S7A and B. Note that these strains are not sensitive to DNA damaging treatments.
(TIF)

Table S1 Rad9, Chk1 activation domain phosphorylation sites assigned by manual interpretation of collision-activated dissociation and/or electron transfer dissociation mass spectra. ^a^ Phosphopeptide levels are reported as % total peptide abundance detected, (++++) = 70–100%, (+++) 35–69%, (++) = 6–34%, (+) = 1–5%, (−) no phosphorylated peptide detected. % Total abundances were calculated from ion currents observed in the main beam mass spectra [(phosphorylated peptide ion current)/(phosphorylated peptide+non-phosphorylated peptide ion current)×(100)]. ^b^ Phosphorylated peptide containing this phosphorylation site was assigned by HPLC retention time and correct accurate mass, only. ^c^ ND = Not detected. Non-phosphorylated and phosphorylated peptides were not detected.
(DOCX)

Table S2 Strains used in this study.
(DOCX)

Table S3 Plasmids used in this study.
(DOCX)

Table S4 Primers used in this study.
(DOCX)

Table S5 Amino acid sequences of the peptides used in array. Peptides arrays of immobilized overlapping 19-mer peptides, each shifted to the right by 3 amino acids encompassing the entire Rad9 CAD sequence were generated. At position A1, we included amino acids 2–20 of Rad9 (*i.e.* without starting Methionine). For the control peptides, we used peptides containing target sites for Cdc28 (D1 to D9), CK2 (D11 to D15) and Cdc7 kinases (D17 to D20).
(DOCX)

Text S1 Supplemental description of material and methods used in this study.
(DOC)
