# Supplementary figures and images for: Site-Specific Phosphorylation of the DNA Damage Response Mediator Rad9 by Cyclin-Dependent Kinases Regulates Activation of Checkpoint Kinase 1
Source: PLoS Genet. 2013 Apr 4;9(4):e1003310. doi: 10.1371/journal.pgen.1003310 (PMC3616908; doi:10.1371/journal.pgen.1003310)

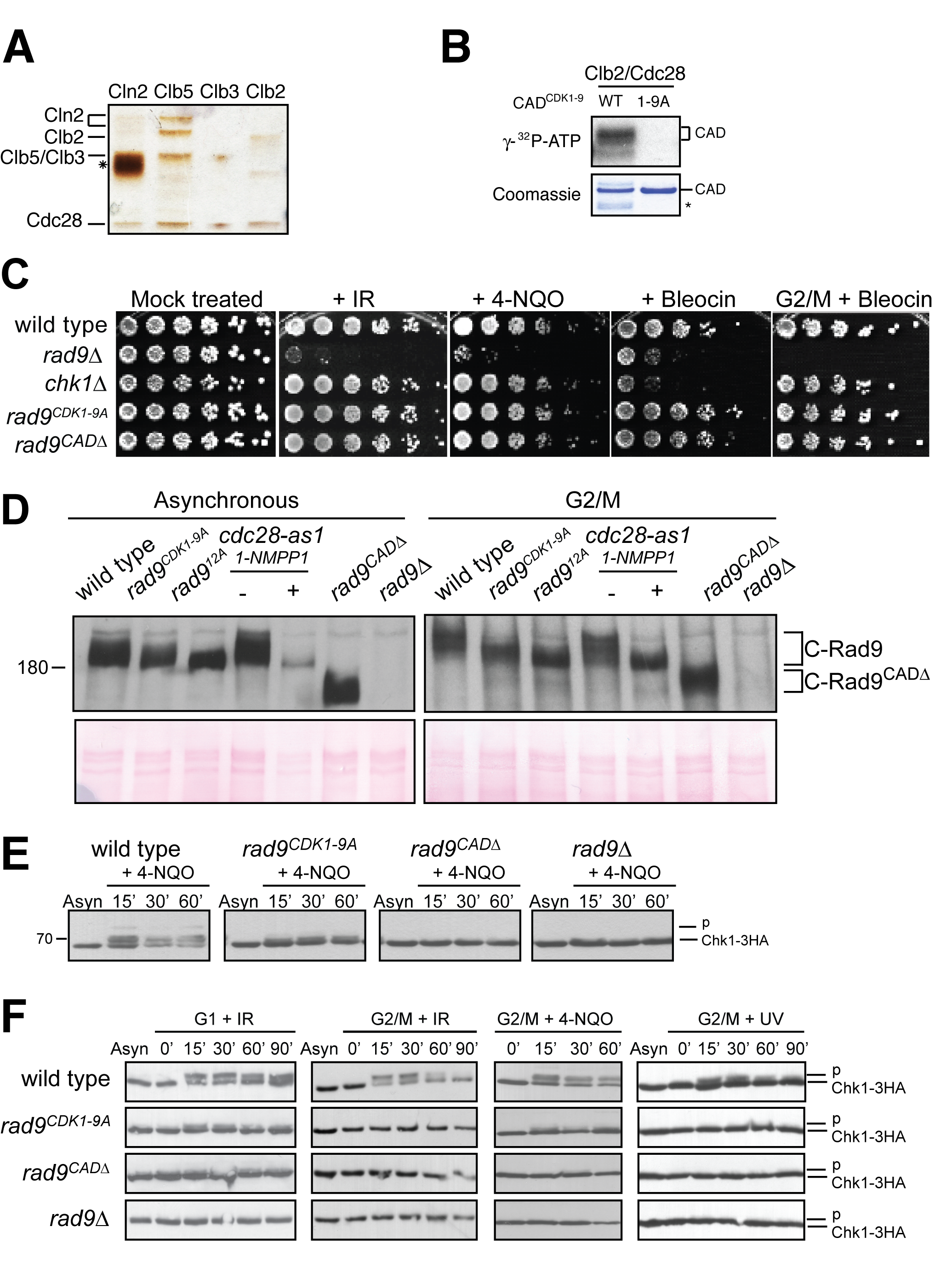

Supplement: Figure S2 — Related to Figure 2. The CDK1-9 sites within the CAD region of Rad9 are phosphorylated both in vitro and in vivo. (A) Silver-stained gel of the purified Cdc28/Cln complexes used in this study. * indicates a contaminant in the Cdc28/Cln2 purification. (B) Cdc28/Clb2 phosphorylates Rad9 CADWT but not CADCDK1-9A in vitro. In vitro kinase assays were performed on the indicated substrates with higher concentration of Cdc28/Clb2 complex than the one presented in Figure 5D (3.6 nM compared to 0.6 nM). * indicates degradation product of Rad9 CADWT. (C) Cells expressing Rad9CDK1-9A as their only Rad9 protein are not sensitive to the indicated DNA damaging treatments. Drop tests were performed in the indicated strains. Note that the bleocin sensitivity of proliferating chk1Δ cells could indicate a role for CHK1 in surviving bleocin-induced lesions during S phase, which can be rescued by a transient arrest at the G2/M transition induced on nocodazole plates. This role is clearly independent from the N terminus of Rad9. (D) Rad9CDK1-9A displays defective cell cycle and Cdc28-dependent phosphorylation in vivo. Rad9 western blot prepared from the indicated strains. Rad912A is the short name of a strain expressing rad9CDK1,3,4,6,9,11,14,16-20A (Karen Finn, Unpublished data). 1-NMPP1 treatment of cdc28-as1 cells was used to indicate Cdc28-dependent phosphorylation. (E) DNA damage-induced Chk1 phosphorylation is defective in rad9CDK1-9A, rad9CADΔ and rad9 Δ cells. Asynchronously growing cells were treated with 4-NQO for the indicated times and Chk1 phosphorylation analysed by western blotting. (F) IR, 4-NQO or UV-induced Chk1 phosphorylation is abolished in nocodazole arrested rad9CDK1-9A, rad9CAD and rad9Δ cells, but there is residual Chk1 activation partially dependent on the CDK1-9 sites in G1-arrested cells. (TIF) [file pgen.1003310.s002.tif]

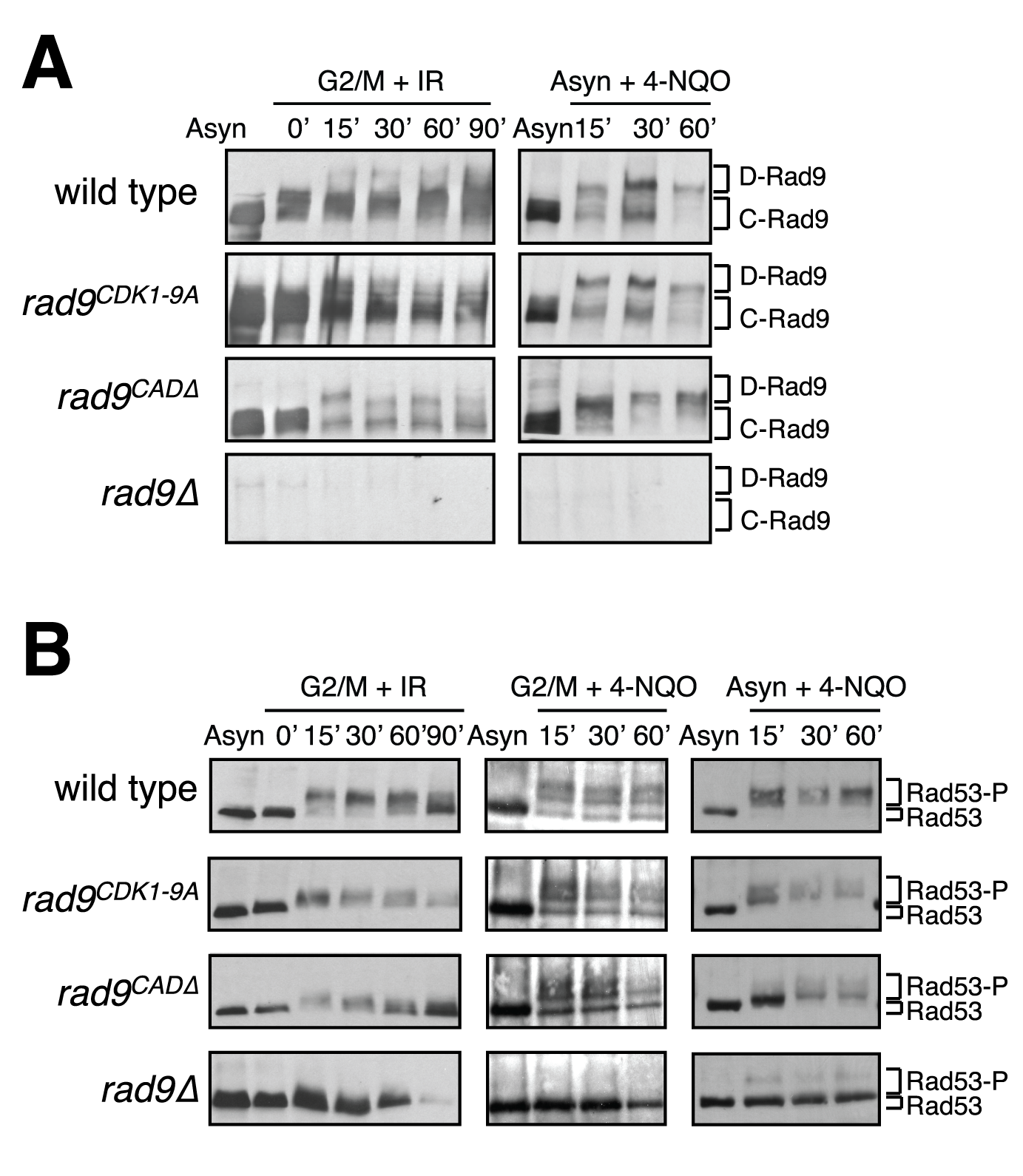

Supplement: Figure S3 — Related to Figure 3. The CDK1-9 sites within the CAD region of Rad9 are not required for damage-induced Rad9 and Rad53 phosphorylations. (A) Rad9 DNA damage-induced phosphorylation is not dependent on the CDK1-9 sites in G2/M-arrested cells after IR and in asynchronously growing cells after 4-NQO. (B) Rad53 DNA damage-induced phosphorylation is not dependent on the CDK1-9 sites in G2/M-arrested cells after IR or 4-NQO and in asynchronous cells after 4-NQO. (TIF) [file pgen.1003310.s003.tif]

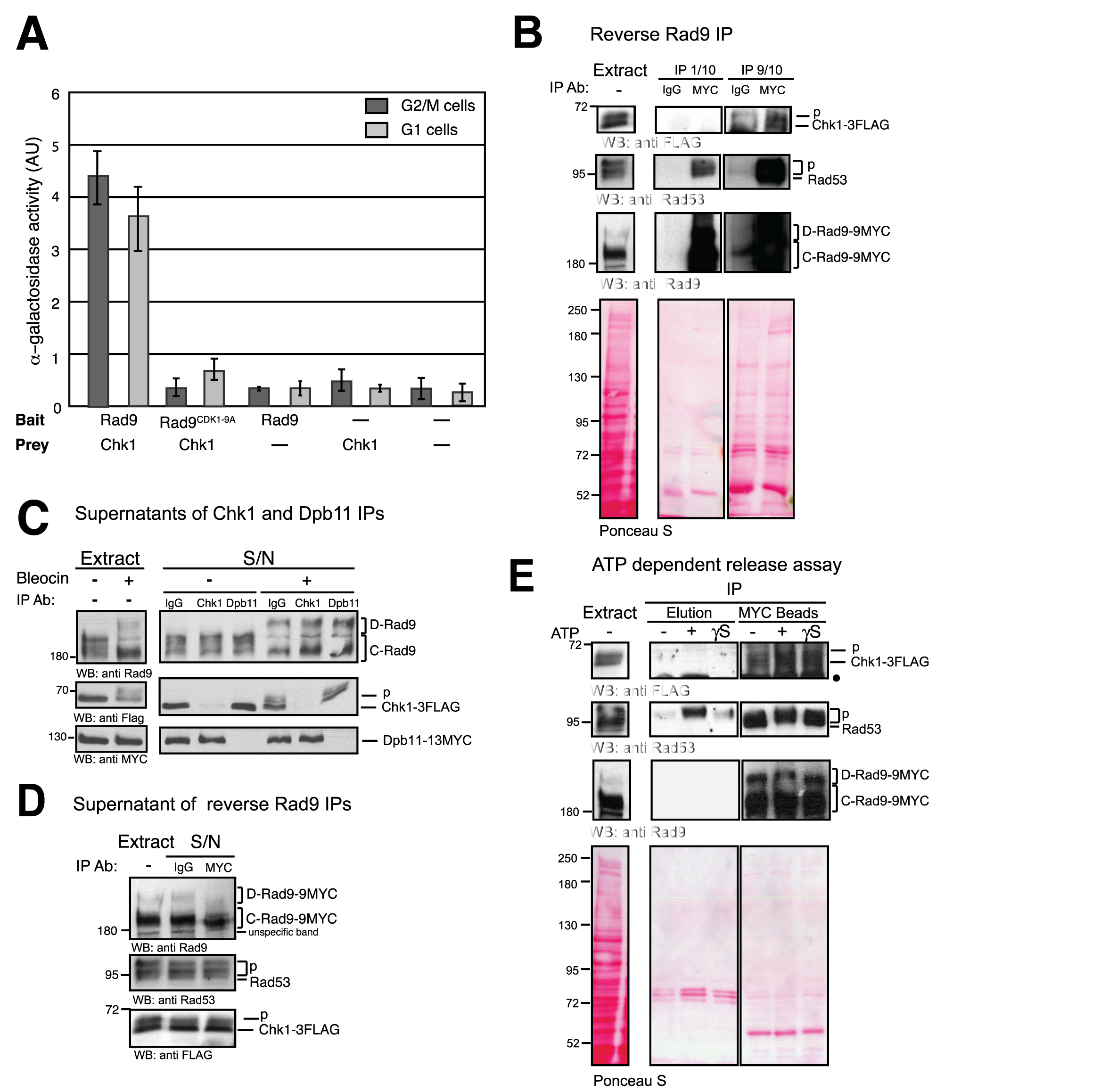

Supplement: Figure S5 — Related to Figure 5. Interaction between Rad9 and Chk1 is dependent on the Rad9 CDK1-9 sites. (A) The Y2H interaction between Rad9 and Chk1 is dependent on the CDK1-9 sites in both G1 and G2/M cells. The indicated bait and prey plasmids introduced into Y2H cells (identical to clones shown in S5A) were grown, divided into two flasks and arrested in G2/M and G1 phases of cell cycle. 1 ml of cells corresponding to one OD value were used to perform the PNP assay (see supplementary information). The α-galactosidase activity was measured according to Clontech Yeast Two Hybrid instructions. (B) Western blotting analysis of the indicated proteins in a reciprocal IP using Rad9-9MYC and Chk1-3FLAG expressing cells confirms the Rad9 and Chk1 interaction. Anti-MYC antibodies were used with extracts from nocodazole-arrested cells, treated with 20 µg/ml of bleocin for 45 min and a mock (IgG) control was performed. Rad9 binding to Rad53 was used as a further control. Different exposures of the crude extracts and the IPs lanes are shown to allow visualization of Rad9-9MYC, Chk1-3FLAG and Rad53 specific bands. (C) Western blotting analysis of the supernatants resulting from the IP experiment using Chk1-3FLAG and Dpb11-13MYC expressing cells presented in Figure 5C. (D) Western botting analysis of the supernatants resulting from IP experiment using Rad9-9MYC and Chk1-3FLAG expressing cells presented in Figure S5B. (E) ATP-dependent release of Rad53, but not Chk1 from Rad9 IPs. Assays were performed as described (Gilbert et al, 2001) except Rad9-9MYC was immunoprecipitated using an anti-MYC monoclonal antibody. The extract was prepared from nocodazole-arrested cells, treated with 20 µg/ml of bleocin for 45 min that expressed both Rad9-9MYC and Chk1-3FLAG. The amount of Rad9-9MYC, Chk1-3FLAG or Rad53 remaining on the beads (Beads) or released (Elution) after incubation with ATP (+), ATP-γS (γS), a non-hydrolysable analogue, or mock treatment without any nucleotide (−) was determined by [file pgen.1003310.s005.tif]

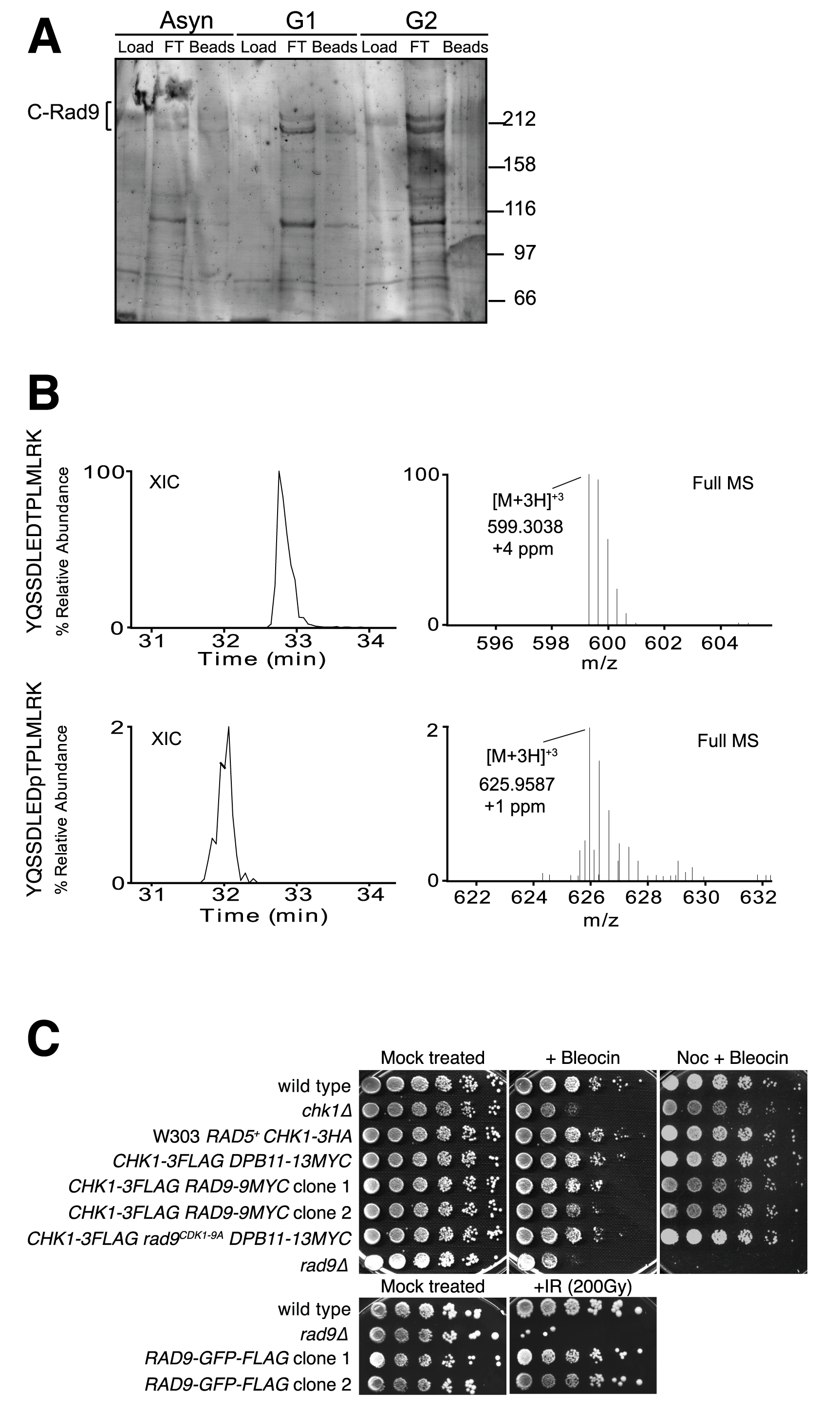

Supplement: Figure S7 — In vivo analysis of Rad9 Chk1 Activation Domain phosphorylation status. (A) Silver stained gel of the purified Rad9-GFP-FLAG used in ETD-Mass spectrometry analyses shown in Figure 6D and Table S1. This gel shows the result of the second immunopurification step (GFP) described in supplemental methods. The Flag elution (Load) was incubated with GFP-Trap beads. The Rad9-GFP-FLAG retained on these beads (Beads) was used for the analyses. The unbound fraction (FT) shows the presence of two unspecific high molecular weight bands not detected in Rad9 western blotting. (B) Extracted Ion Chromatograms (XIC) and Main Beam Mass Spectra (MS) obtained for the T143-containing Rad9 peptides isolated from G2-arrested cells (YQSSDLEDTPLMLRK – Top Panel and YQSSDLEDpTPLMLRK – Bottom Panel). Signal to noise levels for this phosphopeptide and all others in Table S1 are >100 (S/N = >100). (C) Drop test analysis of the indicated tagged strains used for immunoprecipitation experiments shown in Figure 5 and Rad9 purification used for mass spectrometry analyses in Figure 6D, Table S1 and Figure S7A and B. Note that these strains are not sensitive to DNA damaging treatments. (TIF) [file pgen.1003310.s007.tif]
