# Supplementary material for: Site-Specific Phosphorylation of the DNA Damage Response Mediator Rad9 by Cyclin-Dependent Kinases Regulates Activation of Checkpoint Kinase 1
Source: PLoS Genet. 2013 Apr 4;9(4):e1003310. doi: 10.1371/journal.pgen.1003310 (PMC3616908; doi:10.1371/journal.pgen.1003310)
Supplement: Table S1 — Rad9, Chk1 activation domain phosphorylation sites assigned by manual interpretation of collision-activated dissociation and/or electron transfer dissociation mass spectra. a Phosphopeptide levels are reported as % total peptide abundance detected, (++++) = 70–100%, (+++) 35–69%, (++) = 6–34%, (+) = 1–5%, (−) no phosphorylated peptide detected. % Total abundances were calculated from ion currents observed in the main beam mass spectra [(phosphorylated peptide ion current)/(phosphorylated peptide+non-phosphorylated peptide ion current)×(100)]. b Phosphorylated peptide containing this phosphorylation site was assigned by HPLC retention time and correct accurate mass, only. c ND = Not detected. Non-phosphorylated and phosphorylated peptides were not detected. (DOCX) [file pgen.1003310.s008.docx]

**Table S1 – Rad9, Chk1 activation domain phosphorylation sites assigned by manual interpretation of collision-activated dissociation and/or electron transfer dissociation mass spectra.**

| **Relative Abundance of Individual Peptides**^a^ | | |  |  |  |
| --- | --- | --- | --- | --- | --- |
| **Residues** | **Peptide sequence** | **Phosphorylation site** | **G1** | **G2** | **Asyn** |
| **Mono Phosphorylation** | | | | | |
| 10-21 | SSPDRVTQSAIK | S11 | ++ | +++ | ++ |
| 2-21 | SGQLVQWKSSPDRVTQSAIK | S11 | ++++ ^b^ | ++++ | ++++ |
| 22-46 | EALHSPLADGDMNEMNVPVDPLENK | S26 | ++ | ++ | ++ |
| 47-58 | VNSTNIIEGSPK | S56 | ++ | +++ | +++ |
| 47-65 | VNSTNIIEGSPKANPNPVK | S56 | ++++ | ++++ | ++++ |
| 76-85 | SLGLLDESPR | S83 | + | ++ | ND^c^ |
| 76-109 | SLGLLDESPRHDDELNIEVGDNDRPNANILHNERT | S83 | ++ | ND^c^ | ND^c^ |
| 122-134 | SNRTPGKENLLTK | S125 | ++++ | ++++ | ++++ |
| 135-149 | YQSSDLEDTPLMLRK | S137/T143 | +^b^/+^b^ | +/+ | +^b^/+ |
| 150-163 | KMTFQTPTDPLEQK | T152/T155 | +^b^/+^b^ | +^b^/+^b^ | +/+ |
| 205-220 | SADNYDCALEGIVTPK | S205/T218 | +/++ | +/++ | ND^c^ |
| **Di Phosphorylation** | | | | | |
| 2-21 | SGQLVQWKSSPDRVTQSAIK | S10 & S11/ S11 & T16 | ++/- | ++/+ | ++/- |
| 205-220 | SADNYDCALEGIVTPK | S205 & T218 | + ^b^ | + | ND^c^ |
