# Supplementary material for: Site-Specific Phosphorylation of the DNA Damage Response Mediator Rad9 by Cyclin-Dependent Kinases Regulates Activation of Checkpoint Kinase 1
Source: PLoS Genet. 2013 Apr 4;9(4):e1003310. doi: 10.1371/journal.pgen.1003310 (PMC3616908; doi:10.1371/journal.pgen.1003310)
Supplement: Table S2 — Strains used in this study. (DOCX) [file pgen.1003310.s009.docx]

**Table S2: Strains used in this study.**

| **Collection Number** | **Strain name** | **Relevant Genotype** | **Source** | **Figure** |
| --- | --- | --- | --- | --- |
| **YNL0420** | CG378 | ***MAT*a** *ade5-7 can1-1 leu2-3,112, trp1-289 ura3-52* | L. Johnston | 1A |
| **YNL0220** | W303-1a | ***MAT*a** *ade2-1 trp1-1 leu2-3,112, his3-11,15 ura3-1 can1-100 rad5-535* | K. Nasmyth | 6D, S1A; S6H |
| **YNL1506** | *RAD9-GFP-FLAG,* clone 2 | W303-1a*, RAD9-GFP-FLAG KANMX* | This study | 6D; S6F |
| **YNL1312** | *cdc28-as1* | W303-1a *cdc28-as1 (*JAU01) | ([Bishop et al., 2000](#_ENREF_1)) | 1B; 4, S4, 5B, 6A |
| **YNL0620** | *cdc4-1* | W303-1a *cdc4-1* | J. Diffley | 1C |
| **YNL0264** | *cdc7-1* | W303-1a *cdc7-1* | J. Diffley | 1C |
| **YNL0742** | YLD1 | ***MAT*a** *cdc15-2 cdc6::hisG trp1::TRP1:MET3-CDC6* | J Diffley | S1B |
| **YNL1287** | W303-1a *RAD5^+^* | ***MAT*a** *ade2-1 trp1-1 leu2-3,112, his3-11,15 ura3-1 can1-100 RAD5^+^* | This study | S2C-D |
| **YNL1350** | *rad9^CAD∆^* | W303-1a *RAD5^+^ rad9^CAD∆^* | This study | S2C-D |
| **YNL1347** | *rad9^CDK1-9A^* | W303-1a *RAD5^+^ rad9^S11A,S26A,S56A,S83A,T110A,T125A,T143A,T155A,T218A^* | This study | S2C-D |
| **YNL1016** | *rad9∆* | W303-1a *rad9::URA3* | ([Grenon et al., 2007](#_ENREF_12)) | S2C-D, S6H |
| **YNL1145** | *sml1∆ chk1∆* | W303-1a *RAD5^+^ sml1::KanMX6 chk1::URA3* | This study | S2C; 3C; S6H |
| **YNL1280** | *CHK1-3HA* | W303-1a *CHK1-3HA::KlURA3* | ([Clerici et al., 2004](#_ENREF_3)) |  |
| **YNL1144** | *CHK1-3HA RAD5^+^* | W303-1a *RAD5^+^ CHK1-3HA::KlURA3* | This study | 2C-E; 3A-B S2E-F; S3;S5B; S6H |
| **YNL1431** | *rad9^CDK1-9A^ CHK1-3HA* | W303-1a *RAD5^+^* *rad9^Cdk1-9A^ CHK1-3HA::KlURA3* | This study | 2C-E; 3A-B S2E-F; S3; S6B |
| **YNL1432** | *rad9^CAD∆^ CHK1-3HA* | W303-1a *RAD5^+^* *rad9^CAD∆^ CHK1-3HA::KlURA3* | This study | 2C-E; 3A-B S2E-F; S3; S6B |
| **YNL1433** | *rad9∆ CHK1-3HA* | W303-1a *RAD5^+^* *rad9::LEU2 CHK1-3HA::KlURA3* | This study | 2C-E; 3A-B S2E-F; S3; S6B |
| **YNL1445** | *sml1∆ rad53∆ chk1∆* | ***MAT*a**  *sml1:: kanMX6 rad53::HIS3 chk1::URA3* *RAD5^+^* *ade2-1 trp1-1 can1-100 ura3* | ([Blankley and Lydall, 2004](#_ENREF_2)) | 3C |
| **YNL1143** | *sml1∆* | W303-1a *RAD5^+^* *sml1::KanMX6* | This study | 3C |
| **YNL1447** | *sml1∆ rad53∆* | W303-1a *RAD5^+^ sml1::KanMX6 rad53::HIS3* | This study | 3C |
| **YNL1142** | *sml1∆ rad53∆ chk1∆* | W303-1a *RAD5^+^ sml1::KanMX6 rad53::HIS3 chk1::URA3* | This study | 3C |
| **YNL1146** | *sml1∆ rad9^CDK1-9A^* | W303-1a *RAD5^+^ sml1::KanMX6 rad9^CDK1-9A^* | This study | 3C |
| **YNL1147** | *sml1∆ rad9^CDK1-9A^ chk1∆* | W303-1a *RAD5^+^ sml1::KanMX6 rad9^CDK1-9A^ chk1::URA3* | This study | 3C |
| **YNL1240** | *sml1∆ rad9^CDK1-9A^ rad53∆* | W303-1a *RAD5^+^* *sml1::KanMX6 rad9^CDK1-9A^ rad53::HIS3* | This study | 3C |
| **YNL1239** | *sml1∆ rad9^CDK1-9A^ rad53∆ chk1∆* | W303-1a *RAD5^+^* *sml1::KanMX6 rad9^CDK1-9A^ rad53::HIS3 chk1::URA3* | This study | 3C |
| **YNL1441** | *rad9^CDK1,3,4,6,9A^ CHK1-3HA* | W303-1a *RAD5^+^ rad9^S11A,S56A,S83A,T125A,T218A^ CHK1-3HA::KlURA3* | This study | S6B |
| **YNL1440** | *rad9^CDK4,6,9A^ CHK1-3HA* | W303-1a *RAD5^+^ rad9^S83A,T125A,T218A^ CHK1-3HA::KlURA3* | This study | S6B |
| **YNL1419** | *rad9^CDK5A^ CHK1-3HA* | W303-1a *RAD5^+^ rad9^T110A^ CHK1-3HA::KlURA3* | This study | S6B |
| **YNL1439** | *rad9^CDK7,8,9A^ CHK1-3HA* | W303-1a *RAD5^+^* *rad9^T143A,T155A,T218A^ CHK1-3HA::KlURA3* | This study | S6B |
| **YNL1443** | *rad9^CDK1,2,PIK1A^ CHK1-3HA* | W303-1a *RAD5^+^* *rad9 ^S11A,S26A,T16A^ CHK1-3HA::KlURA3* | This study | S6B |
| **YNL1435** | *rad9^CDK1A^ CHK1-3HA* | W303-1a *RAD5^+^* *rad9 ^S11A^ CHK1-3HA::KlURA3* | This study | S6B |
| **YNL1437** | *rad9^CDK9A^ CHK1-3HA* | W303-1a *RAD5^+^ rad9^T218A^ CHK1-3HA::KlURA3* | This study | S6B |
| **YNL1424** | *cdc28-as1 CHK1-3HA* | W303-1a *cdc28-as1 CHK1-3HA::KlURA3* | This study | 4; S4 |
| **YNL1425** | Y2HGold | *MATa, trp1-901, leu2-3, 112, ura3-52, his3-200, gal4Δ, gal80Δ, LYS2::GAL1UAS–Gal1TATA–His3, GAL2UAS–Gal2TATA–Ade2 URA3::MEL1UAS–Mel1TATA AUR1-C MEL1* | Clontech | 5A |
| **YNL1426** | *DBP11-13MYC* | W303-1a *DPB11-13MYC::HIS3* | ([Puddu et al., 2008](#_ENREF_18)) |  |
| **YNL1427** | *CHK1-3FLAG DBP11-13MYC* | W303-1a *RAD5^+^ CHK1-3FLAG::KANMX DPB11-13MYC::HIS3* | This study | 5C-D; S5C; S6G |
| **YNL1428** | *rad9^CDK1-9^*^A^ CHK1-3FLAG DBP11-13MYC | W303-1a *RAD5^+^* *rad9^CDK1-9A^ CHK1-3FLAG::KANMX DPB11-13MYC::HIS3* | This study | 5C-D; S5C; S6G |
| **YNL1430** | *RAD9-9MYC* | W303-1a *RAD9-9MYC::TRP1* | ([Giannattasio et al., 2002](#_ENREF_7)) |  |
| **YNL1500** | *RAD9-9MYC CHK1-3FLAG* | W303-1a *RAD9-9MYC::TRP1 CHK1-3FLAG::KANMX* | This study | S5B, S5D, S6G |
| **YNL1505** | *rad9∆ CHK1-3FLAG* | W303-1a *RAD5^+^ rad9::HIS3 CHK1-3FLAG::KANMX* | This study | 6B |
| **YNL0351** | DOM0949 | *CLN2-3HA:HISMX6 bar1:hisG* | *(*[*Koivomagi et al., 2011*](#_ENREF_14)*)* | 2B; S2A-B |
| **YNL0350** | DOM0076 | *gal-CLB5-TAP pRSAB1234-URA3 bar1:HISG sic1d::LEU2* | *(*[*Koivomagi et al., 2011*](#_ENREF_14)*)* | 2B; S2A-B |
| **YNL0352** | DOM0957 | *gal-CLB3-TAP pRSAB1234-URA3 bar1:HISG sic1d::LEU2* | *(*[*Koivomagi et al., 2011*](#_ENREF_14)*)* | 2B; S2A-B |
| **YNL0353** | DOM0077 | *gal-CLB2-TAP pRSAB1234-URA3 bar1:HISG sic1d::LEU2* | *(*[*Koivomagi et al., 2011*](#_ENREF_14)*)* | 2B; 6C; S2A-B |
