# Supplementary material for: Site-Specific Phosphorylation of the DNA Damage Response Mediator Rad9 by Cyclin-Dependent Kinases Regulates Activation of Checkpoint Kinase 1
Source: PLoS Genet. 2013 Apr 4;9(4):e1003310. doi: 10.1371/journal.pgen.1003310 (PMC3616908; doi:10.1371/journal.pgen.1003310)
Supplement: Table S3 — Plasmids used in this study. (DOCX) [file pgen.1003310.s010.docx]

**Table S3: Plasmids used in this study.**

| **Collection Number** | **Plasmid** | **Usage** | **Source** |
| --- | --- | --- | --- |
| **ENL0757** | pVF6 | *cdc28-as1* mutation | Diani et al., 2009 |
| **ENL0749** | pRS306-*rad9^CDK1-9A^* | Integrative vector | This study |
| **ENL0026** | pRS306-*rad9^CDK1,3,4,6,9A^* | Integrative vector | This study |
| **ENL0758** | pRS306-*rad9^CDK5A^* | Integrative vector | This study |
| **ENL0746** | pRS306-*rad9^CDK1A^* | Integrative vector | This study |
| **ENL0750** | pRS306-*rad9^CAD∆^* | Integrative vector | This study |
| **ENL0186** | pRS306-CTRAD9 | Integrative vector | This study |
| **ENL0760** | p3FLAG-KANMX | Tagging vector | Gelbart et al., 2001 |
| **ENL0759** | pGEM-Teasy-NTRAD9 | Mutagenesis vector | This study |
| **ENL0747** | pGEM-Teasy-NT*rad9^CDK1-9A^* | Mutagenesis vector | This study |
| **ENL0011** | pGEM-Teasy-NT*rad9^CDK1,3,4,6,9A^* | Mutagenesis vector | This study |
| **ENL746** | pGEM-Teasy-NT*rad9^CDK1A^* | Mutagenesis vector | This study |
| **ENL0748** | pGEM-Teasy-NT*rad9^CAD∆^* | Mutagenesis vector | This study |
| **ENL0706** | pGEM-Teasy-CTRAD9 | Mutagenesis vector | This study |
| **ENL0587** | pGBKT7-BD | Y2H vector | Clontech |
| **ENL0588** | pGBKT7-Lam | Y2H vector | Clontech |
| **ENL0589** | pGBKT7-53 | Y2H vector | Clontech |
| **ENL0590** | pGBKT7-BD-RAD9 | Y2H vector | This study |
| **ENL0591** | pGBKT7-BD-CAD | Y2H vector | This study |
| **ENL0592** | pGBKT7-BD-*rad9^CDK1-9A^* | Y2H vector | This study |
| **ENL0593** | pGBKT7-BD-CHK1 | Y2H vector | This study |
| **ENL0594** | pGADT7-T | Y2H vector | Clontech |
| **ENL0595** | pGADT7-AD | Y2H vector | Clontech |
| **ENL0596** | pGADT7-AD-RAD9 | Y2H vector | This study |
| **ENL0597** | pGADT7-AD-*rad9^CDK1-9A^* | Y2H vector | This study |
| **ENL0598** | pGADT7-AD-CAD | Y2H vector | This study |
| **ENL0599** | pGADT7-AD-CHK1 | Y2H vector | This study |
| **ENL0600** | pEG202 | Y2H vector | Granata et al., 2010 |
| **ENL0601** | pEG202-CHK1 | Y2H vector | This study |
| **ENL0602** | pJG4-5 | Y2H vector | Granata et al., 2010 |
| **ENL0603** | pJG4-5-RAD9 | Y2H vector | This study |
| **ENL0604** | pSH18-34 | Y2H vector | Granata et al., 2010 |
| **ENL0605** | pJG4-5-CAD^WT^ | Y2H vector | This study |
| **ENL0606** | pJG4-5-CAD^CDK1-9A^ | Y2H vector | This study |
| **ENL0607** | pJG4-5-CAD^CDK1-9A + 1WT^ | Y2H vector | This study |
| **ENL0608** | pJG4-5-CAD^CDK1-9A + 2WT^ | Y2H vector | This study |
| **ENL0609** | pJG4-5-CAD^CDK1-9A + 3WT^ | Y2H vector | This study |
| **ENL0610** | pJG4-5-CAD^CDK1-9A + 4WT^ | Y2H vector | This study |
| **ENL0611** | pJG4-5-CAD^CDK1-9A + 5WT^ | Y2H vector | This study |
| **ENL0612** | pJG4-5-CAD^CDK1-9A + 6WT^ | Y2H vector | This study |
| **ENL0613** | pJG4-5-CAD^CDK1-9A + 7WT^ | Y2H vector | This study |
| **ENL0614** | pJG4-5-CAD^CDK1-9A + 8WT^ | Y2H vector | This study |
| **ENL0615** | pJG4-5-CAD^CDK1-9A + 9WT^ | Y2H vector | This study |
| **ENL0619** | pJG4-5-CAD^CDK6A,7A^ | Y2H vector | This study |
| **ENL0620** | pJG4-5-CAD^CDK1-9A + 6D^ | Y2H vector | This study |
| **ENL0621** | pJG4-5-CAD^CDK1-9A + 7D^ | Y2H vector | This study |
| **ENL0622** | pJG4-5-CAD^CDK1-9A + 6D7D^ | Y2H vector | This study |
| **ENL0623** | pJG4-5-CAD^CDK1-9A + 6WT+7WT^ | Y2H vector | This study |
| **ENL0616** | pet15-CAD^WT^ | Recombinant CAD^WT^ | This study |
| **ENL0617** | pet15-CAD^CDK1-9A^ | Recombinant CAD^CDK1-9A^ | This study |
| **ENL0530** | pFA6a-GFP(S65T)-kanMX6 | C-terminal GFP tagging module | Longtine et al., 1998 |
