# Supplementary material for: Site-Specific Phosphorylation of the DNA Damage Response Mediator Rad9 by Cyclin-Dependent Kinases Regulates Activation of Checkpoint Kinase 1
Source: PLoS Genet. 2013 Apr 4;9(4):e1003310. doi: 10.1371/journal.pgen.1003310 (PMC3616908; doi:10.1371/journal.pgen.1003310)
Supplement: Table S4 — Primers used in this study. (DOCX) [file pgen.1003310.s011.docx]

**Table S4: Primers used in this study.**

| **Name** | ***5’🡪 3’ Sequence*** | **Usage** |
| --- | --- | --- |
| Rad9seq2 | CGGCCTTGTTAGCGTTAGAT | To amplify *CAD* region of *RAD9* from genomic DNA |
| Rad9 cdkwtm2 | GCAAGATAGAGAAACGCCATAG | To amplify *CAD* region of *RAD9* from genomic DNA |
| N1 Mut | GCAAGATAGAGAAACGCCATAG | *CAD* mutants sequencing |
| Chk1-Dia F | CACGTTGCAACCTATGGATG | To amplify *CHK1-3HA::KlURA3* cassette; *CHK1-3FLAG* diagnosis PCR |
| Chk1-Dia R | GAACGGAATATCTGTGGAAG | To amplify *CHK1-3HA:: KlURA3* cassette |
| Chk1-Flag-5’ | AATTTCAACTATCTGTAGGGATATTATCCTAATTCCCAACAGGGAACAAA AGCTGGAG | To amplify *CHK1-3FLAG::KANMX* |
| Chk1-Flag-3’ | TGATCAGTGCATCTTAACCCTTCTTTTGTCTCCATTTTTTCTATAGGGCGAATTGGGT | To amplify *CHK1-3FLAG::KANMX* |
| Chk1-2 | GTTACGATGACACACTAG | *CHK1-3FLAG* diagnosis PCR |
| DPB11-myc-5’ | GAAAAACCTATGAGACGACAGACAAGAAATCAGACAAAGGAATTAGATTCTCGGATCCCCGGGTTAATTAA | To amplify *DPB11-13MYC::HIS3* |
| DPB11-myc-3’ | GCTGTAGATGGCGTATGTAAATGAATATCTTATAAAATTACGGACTACATTTCAGAATTCGAGCTCGTTTAAAC | To amplify *DPB11-13MYC::HIS3* |
| DPB11-testF | AGCTTGAACTCGTGGTTCCT | *DPB11-13MYC* diagnosis PCR |
| DPB11-testR | ATGCCAGGAGGCATAAGGTT | *DPB11-13MYC* diagnosis PCR |
| Rad9N1F | GTTCAATGGAAAAGCGCTCCAGATCGAGTC | Mutagenesis of S11 to Ala |
| Rad9N1R | GACTCGATCTGGAGCGCTTTTCCATTGAAC | Mutagenesis of S11 to Ala |
| Rad9N2F | CATAATCGAAGGAGCTCCCAAAGCAAATCC | Mutagenesis of S56 to Ala |
| Rad9N2R | GGATTTGCTTTGGGAGCTCCTTCGATTATG | Mutagenesis of S56 to Ala |
| Rad9N3F | GGATTACTTGACGAGGCTCCAAGACATGATG | Mutagenesis of S83 to Ala |
| Rad9N3R | CATCATGTCTTGGAGCCTCGTCAAGTAATCC | Mutagenesis of S83 to Ala |
| Rad9N4F | CAAAAGCAATCGAGCCCCTGGTAAAG | Mutagenesis of T125 to Ala |
| Rad9N4R | CTTTACCAGGGGCTCGATTGCTTTTG | Mutagenesis of T125 to Ala |
| Rad9N5F | GCATTGGAAGGAATTGTTGCACCTAAAAG | Mutagenesis of T218 to Ala |
| Rad9N5R | CTTTTAGGTGCAACAATTCCTTCCAATGC | Mutagenesis of T218 to Ala |
| R9T110A | TAACATATTGCATAATGAAAGGGCTCCTGACCTTGACCGAATTG | Mutagenesis of T110 to Ala |
| R9S26A | TATAAAGGAAGCACTGCATGCTCCCTTGGCTGATGGCGAC | Mutagenesis of S26 to Ala |
| R9N1T16A | AAGCGCTCCAGATCGAGTCGCCCAAAGCGCTATAAAGGAAG | Mutagenesis of T16 to Ala |
| R9T155A | GAAAAAAAATGACTTTTCAAGCTCCAACTGATCCATTGGAAC | Mutagenesis of T155 to Ala |
| R9T143A | CAAAGCTCCGATCTGGAAGACGCTCCTCTGATGTTAAGAAAAAAAATG | Mutagenesis of T143 to Ala |
| CADP1 | CGCGGATCCAGTAGAACACTTGGGAATCC | To generate pGEMTeasy-NT*rad9^CAD∆^* |
| CADP2 | CTCGTATCACTCTTTTAGAAGTTGTAGTCCCGATACGTTCTACTTGCTCAAG | To generate pGEMTeasy-NT*rad9^CAD∆^* |
| CADP3 | GAAAATCTTCAACATCAGGG*CT*ATGCAAGATGAACGAGTTCAAAAAACTCAAATC | To generate pGEMTeasy-NT*rad9^CAD∆^* |
| CADP4 | CTTCTGTCTGGCCAGTAGAATGAAAAACAGG | To generate pGEMTeasy-NT*rad9^CAD∆^* |
| pGBKT7/pGADT7-AD-RAD9F | CATGCACATATGTCAGGCCAGTTAGTTCAATGGAAAAGC | Y2H *RAD9* constructs |
| pGBKT7/pGADT7-AD-RAD9R | CATGACGGATCCCTCATCTAACCTCAGAAATAGTGTTG | Y2H *RAD9* constructs |
| pGBKT7/pGADT7-AD-CHK1F | CATGCACATATGAGTCTCTCGCAGGTGTCACCTTTACC | Y2H *CHK1* constructs |
| pGBKT7/pGADT7-AD-CHK1R | CATGACGAATTCTCAGTTGGGAATTAGGATAATATCC | Y2H *CHK1* constructs |
| pGBKT7/pGADT7-AD-RAD9CADR | CATGACGGATCCTCATCCTCCACTTTTACTTAATTCGTC | Y2H *CAD* constructs |
| pEG202CHK1F | CATGCAGAATTCATGAGTCTCTCGCAGGTGTCACCTTTACC | To amplify *CHK1* in Y2H constructs |
| pEG202CHK1R | CATGACCTCGAGTCAGTTGGGAATTAGGATAATATCC | To amplify *CHK1* in Y2H constructs |
| T7 Promoter | GTAATACGACTCACTATAGGGCGA | To sequence Y2H constructs |
| Rad9 Seq4 | GTTGAAGTCAGATACTGGG | *RAD9* sequencing |
| Rad9 Seq6 | TTTCCCAAGGCATATCTGC | *RAD9* sequencing |
| Rad9 Seq8 | CGAAAGCAAAGGACAGAGC | *RAD9* sequencing |
| Rad9 Seq10 | GGATTCTAGAGACGCATTAGC | *RAD9* sequencing |
| Rad9 Seq12 | GGTAAATCTCAGATGAAGC | *RAD9* sequencing |
| Chk1CD+600NtsF | ATGGATCAAAGGGGTTCTCCAC | *CHK1* sequencing |
| pGAD-AD-R | AGATGGTGCACGATGCACAG | *RAD9/CHK1* Y2H vector sequencing |
| pGBK-BD-R | TTTTCGTTTTAAAACCTAAGAGTC | *RAD9/CHK1* Y2H vector sequencing |
| AFG476 | GGCCGGCATATGTCAGGCCAGTTAGTTC | Cloning of CAD into pET-15b |
| 1AGFP-BamHI FW | GGTCGACGGATCCCCGGGTTAATTAAC | To generate Rad9-GFP-FLAG PCR cassette |
| 1BGFP-FLAG Rev | GTCGTCGTCGTCCTTGTAATCGCCAGCGGCGCTGCCGTGTTTGTATAGTTCATCCATGCCATGTG | To generate Rad9-GFP-FLAG PCR cassette |
| 2AGFP-FLAG FW | GATTACAAGGACGACGACGACAAGAGCGCTGCCTCTGGCTAGGGCGCGCCACTTCTAAATAAGC | To generate Rad9-GFP-FLAG PCR cassette |
| 2BEcoRI pFA6a rev | CGATGAATTCGAGCTCGTTTAAACTG | To generate Rad9-GFP-FLAG PCR cassette |
| Rad9-PML-FW | GAGGACACTGGTTTTCACGATGATATTACGGACAATGATATATACAACACTATTTCTGAGGTTAGACGGATCCCCGGGTTAATTAA | For chromosomal integration of Rad9-GFP-FLAG PCR cassette |
| Rad9-PML-Rev | CTAAATTTTTTTTTATTTAATCGTCCCTTTCTATCAATTATGAGTTTATATATTTTTATAATTGAATTCGAGCTCGTTTAAAC | For chromosomal integration of Rad9-GFP-FLAG PCR cassette |
