# Supplementary material for: Site-Specific Phosphorylation of the DNA Damage Response Mediator Rad9 by Cyclin-Dependent Kinases Regulates Activation of Checkpoint Kinase 1
Source: PLoS Genet. 2013 Apr 4;9(4):e1003310. doi: 10.1371/journal.pgen.1003310 (PMC3616908; doi:10.1371/journal.pgen.1003310)
Supplement: Table S5 — Amino acid sequences of the peptides used in array. Peptides arrays of immobilized overlapping 19-mer peptides, each shifted to the right by 3 amino acids encompassing the entire Rad9 CAD sequence were generated. At position A1, we included amino acids 2–20 of Rad9 (i.e. without starting Methionine). For the control peptides, we used peptides containing target sites for Cdc28 (D1 to D9), CK2 (D11 to D15) and Cdc7 kinases (D17 to D20). (DOCX) [file pgen.1003310.s012.docx]

**Table S5:** amino acid sequences of the peptides used in array

Peptides arrays of immobilized overlapping 19-mer peptides, each shifted to the right by 3 amino acids encompassing the entire Rad9 sequence were generated. At position A1, we included amino acids 2-20 of Rad9 (i.e. without starting Methionine). For the control peptides, we used peptides containing target sites for Cdc28 (D1 to D9), CK2 (D11 to D15) and Cdc7 kinases (D17 to D20).

| **Grid position** | **Peptide Sequences** | **Comment** |
| --- | --- | --- |
| **A1**  **A2**  **A3**  **A4**  **A5**  **A6**  **A7**  **A8**  **A9**  **A10**  **A11**  **A12**  **A13**  **A14**  **A15**  **A16**  **A17**  **A18**  **A19**  **A20**  **A21**  **A22**  **A23**  **A24**  **A25**  **A26**  **A27**  **A28**  **A29**  **A30**  **B1**  **B2**  **B3**  **B4**  **B5**  **B6**  **B7**  **B8**  **B9**  **B10**  **B11**  **B12**  **B13**  **B14**  **B15**  **B16**  **B17**  **B18**  **B19**  **B20**  **B21**  **B22**  **B23**  **B24**  **B25**  **B26**  **B27**  **B28**  **B29**  **B30**  **C1**  **C2**  **C3**  **C4**  **C5**  **C6**  **C7**  **C8**  **C9**  **C10**  **C11**  **C12**  **C13**  **C14**  **C15**  **C16**  **C17**  **C18**  **C19**  **C20**  **C21**  **C22**  **C23**  **D1**  **D2**  **D3**  **D4**  **D5**  **D6**  **D7**  **D8**  **D9**  **D10**  **D11**  **D12**  **D13**  **D14**  **D15**  **D16**  **D17**  **D18**  **D19**  **D20** | S-G-Q-L-V-Q-W-K-S-S-P-D-R-V-T-Q-S-A-I  M-S-G-Q-L-V-Q-W-K-S-S-P-D-R-V-T-Q-S-A  Q-L-V-Q-W-K-S-S-P-D-R-V-T-Q-S-A-I-K-E  Q-W-K-S-S-P-D-R-V-T-Q-S-A-I-K-E-A-L-H  S-S-P-D-R-V-T-Q-S-A-I-K-E-A-L-H-S-P-L  D-R-V-T-Q-S-A-I-K-E-A-L-H-S-P-L-A-D-G  T-Q-S-A-I-K-E-A-L-H-S-P-L-A-D-G-D-M-N  A-I-K-E-A-L-H-S-P-L-A-D-G-D-M-N-E-M-N  E-A-L-H-S-P-L-A-D-G-D-M-N-E-M-N-V-P-V  H-S-P-L-A-D-G-D-M-N-E-M-N-V-P-V-D-P-L  L-A-D-G-D-M-N-E-M-N-V-P-V-D-P-L-E-N-K  G-D-M-N-E-M-N-V-P-V-D-P-L-E-N-K-V-N-S  N-E-M-N-V-P-V-D-P-L-E-N-K-V-N-S-T-N-I  N-V-P-V-D-P-L-E-N-K-V-N-S-T-N-I-I-E-G  V-D-P-L-E-N-K-V-N-S-T-N-I-I-E-G-S-P-K  L-E-N-K-V-N-S-T-N-I-I-E-G-S-P-K-A-N-P  K-V-N-S-T-N-I-I-E-G-S-P-K-A-N-P-N-P-V  S-T-N-I-I-E-G-S-P-K-A-N-P-N-P-V-K-F-M  I-I-E-G-S-P-K-A-N-P-N-P-V-K-F-M-N-T-S  G-S-P-K-A-N-P-N-P-V-K-F-M-N-T-S-E-I-F  K-A-N-P-N-P-V-K-F-M-N-T-S-E-I-F-Q-K-S  P-N-P-V-K-F-M-N-T-S-E-I-F-Q-K-S-L-G-L  V-K-F-M-N-T-S-E-I-F-Q-K-S-L-G-L-L-D-E  M-N-T-S-E-I-F-Q-K-S-L-G-L-L-D-E-S-P-R  S-E-I-F-Q-K-S-L-G-L-L-D-E-S-P-R-H-D-D  F-Q-K-S-L-G-L-L-D-E-S-P-R-H-D-D-E-L-N  S-L-G-L-L-D-E-S-P-R-H-D-D-E-L-N-I-E-V  L-L-D-E-S-P-R-H-D-D-E-L-N-I-E-V-G-D-N  E-S-P-R-H-D-D-E-L-N-I-E-V-G-D-N-D-R-P  R-H-D-D-E-L-N-I-E-V-G-D-N-D-R-P-N-A-N  D-E-L-N-I-E-V-G-D-N-D-R-P-N-A-N-I-L-H  N-I-E-V-G-D-N-D-R-P-N-A-N-I-L-H-N-E-R  V-G-D-N-D-R-P-N-A-N-I-L-H-N-E-R-T-P-D  N-D-R-P-N-A-N-I-L-H-N-E-R-T-P-D-L-D-R  P-N-A-N-I-L-H-N-E-R-T-P-D-L-D-R-I-A-N  N-I-L-H-N-E-R-T-P-D-L-D-R-I-A-N-F-F-K  H-N-E-R-T-P-D-L-D-R-I-A-N-F-F-K-S-N-R  R-T-P-D-L-D-R-I-A-N-F-F-K-S-N-R-T-P-G  D-L-D-R-I-A-N-F-F-K-S-N-R-T-P-G-K-E-N  R-I-A-N-F-F-K-S-N-R-T-P-G-K-E-N-L-L-T  N-F-F-K-S-N-R-T-P-G-K-E-N-L-L-T-K-Y-Q  K-S-N-R-T-P-G-K-E-N-L-L-T-K-Y-Q-S-S-D  R-T-P-G-K-E-N-L-L-T-K-Y-Q-S-S-D-L-E-D  G-K-E-N-L-L-T-K-Y-Q-S-S-D-L-E-D-T-P-L  N-L-L-T-K-Y-Q-S-S-D-L-E-D-T-P-L-M-L-R  T-K-Y-Q-S-S-D-L-E-D-T-P-L-M-L-R-K-K-M  Q-S-S-D-L-E-D-T-P-L-M-L-R-K-K-M-T-F-Q  D-L-E-D-T-P-L-M-L-R-K-K-M-T-F-Q-T-P-T  D-T-P-L-M-L-R-K-K-M-T-F-Q-T-P-T-D-P-L  L-M-L-R-K-K-M-T-F-Q-T-P-T-D-P-L-E-Q-K  R-K-K-M-T-F-Q-T-P-T-D-P-L-E-Q-K-T-F-K  M-T-F-Q-T-P-T-D-P-L-E-Q-K-T-F-K-K-L-K  Q-T-P-T-D-P-L-E-Q-K-T-F-K-K-L-K-S-D-T  T-D-P-L-E-Q-K-T-F-K-K-L-K-S-D-T-G-F-C  L-E-Q-K-T-F-K-K-L-K-S-D-T-G-F-C-Y-Y-G  K-T-F-K-K-L-K-S-D-T-G-F-C-Y-Y-G-E-Q-N  K-K-L-K-S-D-T-G-F-C-Y-Y-G-E-Q-N-D-G-E  K-S-D-T-G-F-C-Y-Y-G-E-Q-N-D-G-E-E-N-A  T-G-F-C-Y-Y-G-E-Q-N-D-G-E-E-N-A-S-L-E  C-Y-Y-G-E-Q-N-D-G-E-E-N-A-S-L-E-V-T-E  G-E-Q-N-D-G-E-E-N-A-S-L-E-V-T-E-A-D-A  N-D-G-E-E-N-A-S-L-E-V-T-E-A-D-A-T-F-V  E-E-N-A-S-L-E-V-T-E-A-D-A-T-F-V-Q-M-A  A-S-L-E-V-T-E-A-D-A-T-F-V-Q-M-A-E-R-S  E-V-T-E-A-D-A-T-F-V-Q-M-A-E-R-S-A-D-N  E-A-D-A-T-F-V-Q-M-A-E-R-S-A-D-N-Y-D-C  A-T-F-V-Q-M-A-E-R-S-A-D-N-Y-D-C-A-L-E  V-Q-M-A-E-R-S-A-D-N-Y-D-C-A-L-E-G-I-V  A-E-R-S-A-D-N-Y-D-C-A-L-E-G-I-V-T-P-K  S-A-D-N-Y-D-C-A-L-E-G-I-V-T-P-K-R-Y-K  N-Y-D-C-A-L-E-G-I-V-T-P-K-R-Y-K-D-E-L  C-A-L-E-G-I-V-T-P-K-R-Y-K-D-E-L-S-K-S  E-G-I-V-T-P-K-R-Y-K-D-E-L-S-K-S-G-G-M  V-T-P-K-R-Y-K-D-E-L-S-K-S-G-G-M-Q-D-E  K-R-Y-K-D-E-L-S-K-S-G-G-M-Q-D-E-R-V-Q  K-D-E-L-S-K-S-G-G-M-Q-D-E-R-V-Q-K-T-Q  L-S-K-S-G-G-M-Q-D-E-R-V-Q-K-T-Q-I-M-I  S-G-G-M-Q-D-E-R-V-Q-K-T-Q-I-M-I-S-A-E  M-Q-D-E-R-V-Q-K-T-Q-I-M-I-S-A-E-S-P-N  E-R-V-Q-K-T-Q-I-M-I-S-A-E-S-P-N-S-I-S  Q-K-T-Q-I-M-I-S-A-E-S-P-N-S-I-S-S-Y-D  Q-I-M-I-S-A-E-S-P-N-S-I-S-S-Y-D-K-N-K  I-M-I-S-A-E-S-P-N-S-I-S-S-Y-D-K-N-K-I  **Control peptides**  G-G-A-T-P-K-K-S-A-K-K-T-P-K-K-A-K-K-P  K-K-S-A-K-K-T-P-K-K-A-K-K-P-A-A-A-T-V  Y-P-A-K-L-R-I-P-E-T-P-V-K-K-S-P-L-V-E  H-D-E-V-V-E-I-G-P-T-P-Q-V-Y-G-K-A-I-S  A-I-S-I-F-D-M-N-L-S-P-I-K-P-I-Y-M-T-F  F-D-N-L-R-N-R-S-K-S-P-P-G-F-G-R-L-D-F  T-P-S-P-K-K-N-K-R-S-P-V-K-N-G-G-R-F-T  E-F-L-K-P-P-M-R-I-S-P-N-K-T-D-G-M-K-H  H-I-T-N-I-I-F-P-T-S-P-T-K-L-T-F-S-N-E  (space)  R-R-R-E-E-E-T-E-E-E-E-E-E  S-S-T-S-V-T-P-D-V-S-D-N-E-P-D-H-Y-R-Y  Y-S-K-W-D-K-I-E-L-S-D-D-S-D-V-E-V-H-P  S-G-R-G-K-G-G-K-G-L-G-K-G-G-A-K-R-H-R  G-K-M-V-V-T-G-A-K-S-E-D-D-S-K-L-A-S-R  (space)  L-D-T-S-S-S-S-A-P-P-S-E-A-S-E-P-L-R-I  E-H-S-L-M-I-T-E-T-S-S-P-F-R-S-I-F-S-H  R-D-L-P-P-F-E-D-E-S-E-G-L-L-G-T-E-G-P  R-R-T-D-A-L-T-S-S-P-G-R-D-L-P-P-F-E-D | Amino acids 2-20 of Rad9  Amino acids 1-19 of Rad9  Amino acids 4-22 of Rad9  Amino acids 7-25 of Rad9  Amino acids 10-28 of Rad9  Amino acids 13-31 of Rad9  Amino acids 16-34 of Rad9  Amino acids 19-37 of Rad9  Amino acids 22-40 of Rad9  Amino acids 25-43 of Rad9  Amino acids 28-46 of Rad9  Amino acids 31-49 of Rad9  Amino acids 34-52 of Rad9  Amino acids 37-55 of Rad9  Amino acids 40-58 of Rad9  Amino acids 43-61 of Rad9  Amino acids 46-64 of Rad9  Amino acids 49-67 of Rad9  Amino acids 52-70 of Rad9  Amino acids 55-73 of Rad9  Amino acids 58-76 of Rad9  Amino acids 61-79 of Rad9  Amino acids 64-82 of Rad9  Amino acids 67-85 of Rad9  Amino acids 70-88 of Rad9  Amino acids 73-91 of Rad9  Amino acids 76-94 of Rad9  Amino acids 79-97 of Rad9  Amino acids 82-100 of Rad9  Amino acids 85-103 of Rad9  Amino acids 88-106 of Rad9  Amino acids 91-109 of Rad9  Amino acids 94-112 of Rad9  Amino acids 97-115 of Rad9  Amino acids 100-118 of Rad9  Amino acids 103-121 of Rad9  Amino acids 106-124 of Rad9  Amino acids 109-127 of Rad9  Amino acids 112-130 of Rad9  Amino acids 115-133 of Rad9  Amino acids 118-136 of Rad9  Amino acids 121-139 of Rad9  Amino acids 124-142 of Rad9  Amino acids 127-145 of Rad9  Amino acids 130-148 of Rad9  Amino acids 133-151 of Rad9  Amino acids 136-154 of Rad9  Amino acids 139-157 of Rad9  Amino acids 142-160 of Rad9  Amino acids 145-163 of Rad9  Amino acids 148-166 of Rad9  Amino acids 151-169 of Rad9  Amino acids 154-172 of Rad9  Amino acids 157-175 of Rad9  Amino acids 160-178 of Rad9  Amino acids 163-181 of Rad9  Amino acids 166-184 of Rad9  Amino acids 169-187 of Rad9  Amino acids 172-190 of Rad9  Amino acids 175-193 of Rad9  Amino acids 178-196 of Rad9  Amino acids 181-199 of Rad9  Amino acids 184-202 of Rad9  Amino acids 187-205 of Rad9  Amino acids 190-208 of Rad9  Amino acids 193-211 of Rad9  Amino acids 196-214 of Rad9  Amino acids 199-217 of Rad9  Amino acids 202-220 of Rad9  Amino acids 205-223 of Rad9  Amino acids 208-226 of Rad9  Amino acids 211-229 of Rad9  Amino acids 214-232 of Rad9  Amino acids 217-235 of Rad9  Amino acids 220-238 of Rad9  Amino acids 223-241 of Rad9  Amino acids 226-244 of Rad9  Amino acids 229-247 of Rad9  Amino acids 232-250 of Rad9  Amino acids 235-253 of Rad9  Amino acids 238-256 of Rad9  Amino acids 241-259 of Rad9  Amino acids 242-260 of Rad9  hH1.2  hH1.2  ySwe1-T196  ySld2-T84  ySld2-S100  ySae2-S267  yOrc6-S116S123  yFin1-S54  yFin1-S117  Classical CK2 site  hPTEN CK2site  yCdc37-S14  yhistoneH4-S1  yTBP-S128  yMcm4 5S2S  yMer2 S29S30  hMcm2S53  hMcm2 S40_41 |
