# Supplementary material for: Site-Specific Phosphorylation of the DNA Damage Response Mediator Rad9 by Cyclin-Dependent Kinases Regulates Activation of Checkpoint Kinase 1
Source: PLoS Genet. 2013 Apr 4;9(4):e1003310. doi: 10.1371/journal.pgen.1003310 (PMC3616908; doi:10.1371/journal.pgen.1003310)
Supplement: Text S1 — Supplemental description of material and methods used in this study. (DOC) [file pgen.1003310.s013.doc]

**Abreu – Supplemental Text S1: Supplementary material**

**Itemized list of supplemental material provided:**

**Supplemental material and methods (page 2)**

Yeast strains

Yeast plasmids

Cell cycle and checkpoint experiments

DNA damaging agents

Western blotting and antibodies

Yeast native extracts and immunoprecipitations

Yeast-two hybrid analysis

ATP-dependent release assay

Rad9 dephosphorylation assay

Cdc28 complexes assays

Rad9 peptide pull-down experiments

Mass spectrometry analyses

**Supplemental References (page 19)**

**Supplemental Material and Methods:**

**Yeast Strains:**

All of the strains used in this work, except CG378 used in Figure 1A and the strains used to purify the Cdc28 complexes (Figure 2B and S2A), are in the W303 background and are listed in Table S2. All mutant and tagged alleles used were integrated on the chromosome apart from the yeast two-hybrid experiments. Yeast Strain and plasmid constructions are described below. Plasmids and oligonucleotides used in this study are listed in Tables S3 and S4. The *cdc28-as1* strain was generated by integrating the pVF6 plasmid as described .

**Rad9 mutants:**

Mutations were targeted to the *RAD9* locus using integrative vectors and the final mutant strains generated differed from wild type cells only at the specific *rad9* mutation.

Integrative vectors containing *rad9^CDK1-9A^, rad9^CDK1,3,4,6,9A^, rad9^CDK5A^, rad9^CDK1A^ and rad9^CAD∆^* mutations, respectively, termed pRS306-*rad9^CDK1-9A^,* pRS306-*rad9^CDK1,3,4,6,9A^,* pRS306-*rad9^CDK5A^,* pRS306-*rad9^CDK1A^* and pRS306-*rad9^CAD∆^*, were linearised with MscI and transformed into W303-1a *RAD5^+^* cells. Putative integrants were selected for integration at *RAD9* locus by positive selection on plates lacking uracil. “Pop-out” recombination of the *URA3* marker was achieved by negative selection on 5-fluoroorotic acid (5-FOA). Colonies were screened for the presence of the specific *rad9* mutations by amplifying *RAD9* DNA fragment with Rad9seq2 and Rad9 cdkwtm2 primers and finally sequenced with N1 Mut and other Rad9 sequencing primers (listed in table S4).

Strains used in Figure 3C for epistasis analysis were generated by crossing *MAT*a *rad9^CDK1-9A^* with *MATα sml1::KANMX6 rad53::HIS3 chk1::URA3* (strain DLY2242 in ).

**Chk1 tagged strains:**

To C-terminally tag Chk1 with 3HA, Chk1-Dia F and Chk1-Dia R primers were used to amplify 1.5Kb of *CHK1-3HA*-*KlURA3* DNA cassette from genomic DNA of strain YNL1144 . Wild type and mutant yeast strains were transformed with the *CHK1-3HA*-*KlURA3* DNA cassette and selected for growth on plates lacking uracil. The putative transformants were screened for the expression of Chk1-3HA by western blot analysis and the correct integration of the *CHK1-3HA* allele at the *CHK1* locus was verified by diagnostic PCR.

To C-terminally tag Chk1 with 3-FLAG, Chk1-Flag-5’ and Chk1-Flag-3’ primers were used to amplify a *CHK1-3FLAG-KANMX* DNA cassette from p3FLAG-KanMX plasmid DNA . After positive selection on G418 plates, the putative integrants were screened for the presence of the 3FLAG tag by diagnostic PCR using primers Chk1-Dia F and Chk1-2, and Chk1-3FLAG expression was detected by western blot analysis.

**Dpb11 tagged strains:**

To C-terminally tag Dpb11 with 13MYC, strains were transformed with a *DPB11-13MYC-HIS3* PCR fragment amplified with DPB11-myc-5’ and DPB11-myc-3’ primers from the genomic DNA of strain YNL1426 (strain YFP38 in ). The histidine^+^ integrants were screened for the presence of the 13MYC tag by diagnostic PCR using primers DPB11-testF and DPB11-testR, and Dpb11-13MYC expression was detected by western blot analysis.

**Rad9-GFP-FLAG tagged strain:**

A GFP-FLAG-KANMX C-terminal tagging cassette was created by fusion PCR. Firstly, the GFP coding sequence from the pFA6a-GFP(S65T)-kanMX6 plasmid (Longtine et al, 1998) was fused with the FLAG coding sequence in PCR fragment 1 using both 1A_GFP-BamHI and 1B_GFP-FLAG Rev primers. A second PCR fragment (2) containing 40 bp homology with the FLAG coding sequence 3’ end fused to the KANMX cassette was generated using 2A_GFP-FLAG-FW and 2B_EcoRI-pFA6a primers. The tagging cassette was obtained by PCR fusion of fragment 1 and 2 using the primers 1A_GFP-BamHI and 2B_EcoRI-pFA6a and amplified using long primers bearing 60nt homology to the 3’ end of RAD9 ORF. The resulting fusion PCR product was transformed into W303 and positive clones selected on G418. Correct integration of the GFP-FLAG tag immediately upstream of RAD9 STOP codon was verified by sequencing using Rad9seq12 primer. Wild-type Rad9-GFP-FLAG expression was detected by Rad9, GFP and FLAG western blot analyses. Rad9-GFP-FLAG strains are not sensitive to DNA damage as shown in Figure S7C.

**Yeast Plasmids:**

All constructs were sequenced to confirm the presence and absence of the specific mutations.

**RAD9 integrative plasmids:**

pRS306-*rad9^CDK1-9A^*, pRS306-*rad9^CDK1,3,4,6,9A^*, *pRS306-rad9^CDK1A^,* and *pRS306-rad9^CDK5A^* constructs carrying the specific point mutations were introduced by site-directed mutagenesis (Stratagene) on pGEM-Teasy-NTRAD9, containing 2547 bp fragment of *RAD9* coding for the Rad9 N-terminus, from position –445 to position +2102 within the *RAD9* ORF. Mutations were introduced by successive rounds of mutagenesis using specific sets of primers: Rad9N1F + Rad9N1R; Rad9N2F + Rad9N1R; Rad9N3F + Rad9N3R; Rad9N4F + Rad9N4R; Rad9N5F + Rad9N5R, as well as single primers R9T110A; R9S26A; R9N1T16A; R6T155A and R9T143A.

The pRS306-*rad9^CAD∆^* deletion construct containing a *RAD9* fragment deleted for the sequence coding for the CAD region (from +1 to +693 within the *RAD9* ORF) was generated as follows: A 430 bp fragment named CADA, from position -445 to position -1 from the *RAD9* ORF, was amplified with CADP1 and CADP2 primers. A second 630 bp fragment named CADB, from position +694 to position +2102 within the Rad9 ORF, was amplified with CADP3 and CADP4 primers. CADA and CADB were fused in a 1.107Kb fragment using CADP1 and CADP4 primers and ligated into pGEMTeasy vector by TA cloning (PROMEGA).

The 1.8kb (point mutations) and 1.107Kb (CAD deletion) BamHI-MscI fragments of the resulting pGEM-Teasy-NT*rad9^CDK1-9A^*, pGEM-Teasy-NT*rad9^CDK1,3,4,6,9A^,*pGEM-Teasy-NT*rad9^CDK1A^,* pGEM-Teasy-NT*rad9^CDK5A^* and pGEM-Teasy-*NTrad9^CAD∆^* vectors were cloned into the equivalent sites of the pRS306-NT*rad9^CDK1A^* integrative vector.

**Generation of Y2H constructs:**

The Gal4 binding domain (“Bait”) vectors pGBKT7-BD-RAD9, pGBKT7-BD-CAD, pGBKT7-BD-*rad9^CDK1-9A^* and pGBKT7-BD-CHK1 were generated in two steps. In the first step 3930 bp of *RAD9*, 693 bp coding for CAD, 1584 bp from *CHK1* and 3930bp *rad9^CDK1-9A^* DNA fragments were amplified from the wild type and *rad9^CDK1-9A^* mutant yeast strains by using specific set of primers (pGBKT7+pGADT7-AD-RAD9F and pGBKT7+pGADT7-AD-RAD9R for *RAD9* and *rad9^CDK1-9A^*, pGBKT7+pGADT7-AD-RAD9F and pGBKT7+pGADT7-AD-RAD9CADR for CAD region, pGBKT7+pGADT7-AD-CHK1F and pGBKT7+pGADT7-AD-CHK1R for CHK1) containing restriction sites NdeI-BamHI (for *RAD9*) or NdeI-EcoRI (for *CHK1*). PCR amplified DNA fragments were cloned into pGBKT7 vector. A similar cloning strategy was used to generate the activation domain (“Prey”) vectors pGADT7-AD-RAD9, pGADT7-AD-*rad9^CDK1-9A^,* pGADT7-AD-CAD and pGADT7-AD-CHK1*.* The Y2H RAD9 constructs were sequenced to confirm the desired sequence using specific primers (T7 promoter, Rad9 Seq4, Rad9 Seq6, Rad9 Seq8, Rad9 Seq10, Rad9 Seq12, pGAD-AD-R and pGBK-BD-R). The Y2H CHK1 constructs were sequenced with primers: T7 promoter, Chk1CD+600NtsF, pGAD-AD-R (for AD-CHK1) and pGBK-BD-R (for BD-CHK1).

The Bait plasmid used in the triple plasmid based yeast two hybrid assay expresses a LexA-Chk1 fusion protein and was made as follows: The 1584 bp *CHK1* ORF was amplified from wild type yeast genomic DNA using pEG202CHK1F and pEG202CHK1R primers containg the XhoI and NcoI restriction sites. The XhoI/NcoI fragment was ligated into pEG202 vector cut with the same restriciton enzymes. To generate the pJG4-5-RAD9 , the full length *RAD9* was cloned into pJG4-5 at the XhoI cloning site.

The add-back mutant Y2H constructs were generated as follows: 720 bp DNA fragments encoding the wild type or mutant CAD regions listed below were synthesised by Biomatic Ltd and cloned in the pBMH cloning vector using XhoI and EcoRI flanking cloning sites. The synthesised DNA fragments were subcloned into the pJG4-5 activation domain vector at the XhoI and EcoRI cloning sites to generate the following 7139 bp yeast two-hybrid vectors: *pJG4-5CAD^WT^, pJG4-5CAD^CDK1-9A^, pJG4-5CAD^CDK1-9A+1S^, pJG4-5CAD^CDK1-9A+2S^, pJG4-5CAD^CDK1-9A+3S^, pJG4-5CAD^CDK1-9A+4S^, pJG4-5CAD^CDK1-9A+5T^, pJG4-5CAD^CDK1-9A+6T^, pJG4-5CAD^CDK1-9A+7T^ , pJG4-5CAD^CDK1-9A+8T^, pJG4-5CAD^CDK1-9A+9T^, pJG4-5CAD^CDK1-9A+6T+7T^, pJG4-5CAD^CDK6A,7A^, pJG4-5CAD^CDK1-9A+6D^, pJG4-5CAD^CDK1-9A+7D^, pJG4-5CAD^CDK1-9A+6D+7D^.* The presence of correct mutations was verified by sequencing all inserts using N1-Mut primer before yeast transformation.

The pEG202-CHK1 plasmid DNA was cotransformed with the appropriate pJG4-5 vector and pSH18-34 plasmid and the interaction was analysed as described below.

**CAD^WT^ and CAD^CDK1-9A^ expression vectors:**

The pET-CAD^WT^ and pET-CAD^CDK1-9A^ vectors were generated by amplifying a 717bp PCR fragment coding for CAD^WT^ or CAD^CDK1-9A^ (Rad9 residues 1 to 231) from wild-type genomic DNA and pRS306CDK1-9A, respectively, using primers AFG476 and pGBKT7/pgadt7-ad-rad9cadr. The fragment was digested with NdeI and BamHI and cloned into pET-15b (Novagen, cat. No. 69661-3) predigested with same restriction enzymes. The pET-CAD^WT^ and pET-CAD^CDK1-9A^ plasmids were transformed in the *E. coli* strain Rosetta2 (Novagen). A 50ml cell culture (O.D.=0.8) was treated with 0.4mM IPTG for 4 hours to induce expression of the 10HIS-CAD^WT^ or 10HIS-CAD^CDK1-9A^, and collected by centrifugation. The cell pellet was resuspended in 10 ml of 50mM Tris pH7.5 100mM NaCl and sonicated for one minute at 40% amplitude with 5 second pulses and 10 second intervals with the Branson Sonifier. The lysed samples were loaded for purification on a 1mL His Trap HP column (GE Healthcare) performed in an ÄKTApurifier machine and eluted by gradually adding 50mM Tris, 100mM NaCl, 500mM Imidazole. 1ml fractions were collected and 15µls of each fraction were loaded onto a 12% SDS-PAGE gel for Coomassie staining analysis. Peak fractions were pooled and glycerol was added to a final concentration of 20%. 10µl aliquots of the 10HIS-CADs (approx. 0.35mg/ml) were frozen down to be used in kinase assays.

**Cell cycle and checkpoint experiments**

**G1 arrest and release experiments:**

Asynchronous, exponentially growing cells (5x10^6^ cells/ml) were arrested in G1 phase by addition of α-factor (5 μg/ml final concentration) for 105 minutes. Following arrest, cells were washed with pre-warmed 0.9% saline, followed by YPD, and then resuspended in fresh YPD without α-factor. 2.5x10^7^ cells were taken at the indicated times for western blot, FACS and budding index analyses as previously described .

**CDC mutant experiments:**

Asynchronous, exponentially growing *cdc4-1*, *cdc7-1* and *cdc4-1 sic1 Δ* cells were grown at permissive temperature (25°C) and arrested in G1 as above. 1 hour before removal of α-factor the cells were shifted to the restrictive temperature (37°C) and α-factor removed as above. 2.5x10^7^ cells were taken at the indicated times for western blot and FACS analyses, as previously described .

**Cdc6 experiment:**

Cells in which Cdc6 was either present or absent were released from a late mitotic block (the *cdc15-2*2 mutation at 36^o^C) as described in . Cells were then examined for Rad9 cell cycle phosphorylation and progression through the cell cycle.

**Alpha factor and nocodazole arrest experiments:**

Asynchronous exponentially growing cells (5x10^6^ cells/ml) were arrested either in G1 with 5μg/ml of α-factor or in G2/M with 10μg/ml of nocodazole (Sigma) for 95 minutes. Cells were kept in α-factor or nocodazole for the duration of the experiment. Cells were divided into two and were either mock treated or treated with the indicated DNA damaging agents. For the IR experiment, both treated and untreated samples were resuspended in fresh media containing nocodazole or α-factor after irradiation. Samples were collected at indicated time points for budding index and checkpoint activation analyses .

**G2/M checkpoint analysis:**

G2/M checkpoint activation analysis was performed as reported . G2/M synchronised cells were either mock treated or treated with IR (400Gy), released into medium free of nocodazole but containing  α-factor to trap cycling cells in the subsequent G1 phase. Progression through mitosis was then followed by budding index analysis.

**Cdc28 dependency of checkpoint initiation and maintenance:**

The role of Cdc28 in the initiation of Chk1 phosphorylation was analysed by arresting *cdc28-as1* (analogue sensitive mutant) cells in G2/M with nocodazole. Once arrested, 1-NMPP1 (5μΜ) was added to half of the culture to inhibit Cdc28 activity. After one cell cycle (90 minutes) cells were split into three and were mock treated or damaged with either bleocin (5μg/ml) or 4-NQO (5μΜ). Cell samples were collected at different time points for budding index and western blot analysis.

The role of Cdc28 in the maintenance of Chk1 phosphorylation was analysed by arresting *cdc28-as1* cells with nocodazole. These G2/M arrested cells were mock treated and treated either with bleocin (5μg/ml) or IR (200Gy). After 30 minutes (time 0), 1-NMPP1 (5μΜ) was added to half of the damaged or undamaged cells to inhibit Cdc28 activity. Cells were collected at indicated time points after addition of 1-NMPP1 for the budding index and protein profile analysis.

**CDC phenotype analysis:**

Overnight cultures were grown to a concentration of 5x10^6^ cells/ml. Cultures were divided into two and either mock-treated or irradiated with 200 Gy. Cells were allowed to recover for 3 hours before fixation with 3.7% formaldehyde for 1 hour at room temperature. Cells were subsequently washed with 0.9% saline before imaging. Images were taken with an Axioskop 2 plus microscope (Carl Zeiss MicroImaging, Inc.) coupled to a SPOT Idea 3.0 megapixel digital camera (Diagnostic Instruments, Inc.) and the SPOT software version 4.6.1.3 (Diagnostic Instruments, Inc.). Images were processed using Photoshop and Illustrator software (both Adobe).

**DNA damaging agents**

γ-irradiation (200 or 400 Gy) was carried out using a 137 Cs source at a dose-rate of 12.10 Gy/min (Mainance Engineering, UK). Bleocin (Calbiochem) was used at 0.025 µg/ml for DNA damage sensitivity, 5 µg/ml for checkpoint analyses and 20 µg/ml in the generation of yeast native extracts. 4-NQO (Sigma) was used either at 1.25 µM for DNA damage sensitivity or 5 µM for checkpoint analyses respectively.

DNA damage sensitivity analysis was performed by spotting five-fold serial dilutions (5x10^6^ to 1x10^4^ cells/ml) of exponentially growing cultures of the indicated strains on plates containing the indicated genotoxic agents or treated as indicated. To analyse the DNA damage sensitivity in G2/M arrested cells, exponentially growing cells were arrested in G2/M (for 95 minutes) and spotted as described on YPD agar media containing 1.25µg/ml of nocodazole with and without DNA damaging agents.

**Western blotting and Antibodies**

Sodium hydroxide protein extracts were separated by sodium dodecyl sulphate-polyacrylamide gel electrophoresis (SDS-PAGE). Western blotting was performed as previously described . Rad9, Rad53, Swi6, Dpb11-13MYC, Rad9-9MYC and Chk1-3FLAG were resolved in 6.5%, 80/1 acrylamide/bisacrylamide SDS-PAGE gel and probed with NLO5 , NLO16 or Abcam anti-Rad53 (ab104232) (Figure 3B), NLO2 (D. Lee & N. Lowndes, unpublished) antibodies at 1:10000 dilution, whereas anti-MYC (9E11, Abcam) and anti-FLAG (M2, Sigma) antibodies were used at 1:1000 dilution in PBS containing 0.1% Tween-20.

Sic1, Clb2, Chk1-3HA, CAD-HA and Orc6 were resolved in 10%, 37.5/1 acrylamide/bis-acrylamide, SDS-PAGE gel and probed with anti-Sic1 (JD156 from J. Diffley), anti-Clb2 (sc9071 from Santa Cruz), anti-HA (12CA5) and anti-Orc6 (SB49 from B. Stillman) antibodies at 1:5000, 1:2000, 1:1000, 1:3000 dilutions respectively, in PBS containing 0.1% Tween-20. HRP conjugated anti-mouse secondary antibody (from Pierce) or anti-rabbit secondary antibody (from Pierce) and Super Signal WestPico chemiluminescent substrate from Thermo Scientific (Product no 34080) was used to detect the proteins.

**Yeast two-hybrid analysis:**

**Growth analysis:**

Two-hybrid interactions were assessed by using the Clontech MatchmakerTM Gold Yeast Two-hybrid system (Catalog no 630489). Activation domain vectors (derived from pGADT7-AD) were co-transformed with DNA binding domain vectors (derived from pGBKT7-BD) into the Y2H Gold cells. As a positive control for interaction, co-transformation with pGBKT7-53 and pGADT7-T vectors was performed in Y2H Gold cells. In addition, co-transformation with pGBKT7-Lam and pGADT7-T vectors was performed as a negative control. pGADT7-AD and pGBKT7-BD were also co-transformed, either empty or carrying different combinations of bait and prey proteins, to discriminate auto-activation events. Protein-protein interaction analysis between different bait and prey proteins was assessed by drop test on the SD/-Leu-Trp agar media containing X-alpha-Gal (40mg/ml) and aureobasidin A (125ng/ml) according to the manufacturer’s instructions using six independent clones for each vector combination. Expression of the bait and prey was verified by western blotting.

**G1 and G2/M Yeast Two Hybrid analyses:**

Yeast two hybrid interaction between RAD9^CDK1-9A^ and Chk1 was analysed in G2/M or in G1 using the Clontech MatchmakerTM Gold Yeast Two Hybrid System (Catalog no 630489). Y2H gold cells harboring *pGBKT7rad9^CDK1^*^-9A^ (DNA binding domain vector) and pGADT7-CHK1 (activation domain vector) plasmid DNA were grown in minimal media (SD/-Leu-Trp). Overnight grown cells (corresponding to OD value 1) were arrested in either G2/M or G1 as described earlier. 1 ml of cells was centrifuged at 14000 rpm for 3 minutes and supernatant was collected in a fresh tube. The PNP (Para-Nitrophenyl α-D-Galactopyranoside, Sigma Cat no. N0877) assay was performed with the supernatant and α-galactosidase activity measured according to Clontech Y2H instruction manual. Expression of the bait and prey was verified by western blotting.

**Cdc28- and consensus CDK site- dependency of the Rad9/Chk1 Y2H interaction analyses:**

A triple plasmid-based assay was used to study the Cdc28-dependent interaction between Rad9 and Chk1 proteins as described . Briefly, *cdc28-as1* cells expressing RAD9 prey (pJG4-5-RAD9 or pJG4-5 CAD vectors), CHK1 bait (pEG202-Chk1) and containing the reporter (pSH18-34) were grown overnight in yeast synthetic media (-Ura, -His, -Trp) with 2% (w/v) raffinose (Formedium) to a concentration of 5x10^6^cells/ml. Cells were arrested either in G1 or in G2/M phase as described above. For the Cdc28 dependency experiment, cells were divided into two, one half was mock treated and the other half was treated with 5µM 1-NMPP1 for 1.25 hours to inhibit Cdc28. Galactose (3% w/v) was added into the media to induce *RAD9* expression. After 2 hours, a 15 ml sample was taken, centrifuged and resuspended in 250 µl of breaking buffer (100 mM Tris HCl at pH 8.0, Glycerol 10%; DTT 1 mM, 1 tablet of complete Roche antiproteolytic cocktail). Cells were harvested and ß-galactosidase activity assayed. Cells were lysed by using a Fast-Prep cell disruptor; the optical density (OD) of protein extract at 600 nm was determined by using the Bio-Rad protein assay reagent. 1 ml of Z buffer (60 mM Na2HPO4, 40 mM NaH2PO4, 10 mM KCl, 1 mM MgSO4, and 50 mM ß-mercaptoethanol at pH 7.0) plus ONPG (Ortho 2-Nitrophenyl-ß-D-galactopyranoside) at 4 mg/ml was aliquoted in a small glass tube for each sample. 20 µl of protein extract was added to each tube and incubated at 37°C until a yellow color developed. The reaction was stopped by adding 400 ml of 1 M Na_2_CO_3_ and the OD at 420 nm of each sample was measured. ß-galactosidase activity was calculated using the formula units = 1x10^3^ OD420/(OD600 x reaction time in min). Expression of the bait and prey was verified by measuring the ß-galactosidase activity from cells treated with galactose or not, as well as by western blotting.

**Yeast native extracts and Immunoprecipitations:**

1.5 liter cultures of yeast strains expressing both tagged Chk1-3FLAG and Dpb11-13MYC proteins under the control of their own endogenous promoters either in a wild-type or in *rad9^CDK1-9A^* mutant background were grown in YPD medium at a cell density of 1x10^7^ cells/ml. Cells were then arrested in G2/M phase by addition of 20µg/ml of nocodazole (Sigma) and were either mock treated or treated with 20µg/ml of bleocin (Calbiochem) for 45 minutes. Cells were washed twice with pre-cooled ddH_2_O and once in 2x lysis buffer (300 mM KCl, 100 mM Hepes pH 7.5, 20% glycerol, 8 mM β-mercaptoethanol, 2 mM EDTA, 0.1% Tween20, 0.01% NP-40). Cells were extruded into liquid nitrogen through a syringe and the frozen “noodles” stored at -80ºC until required. Noodles were manually ground in a mortar in liquid nitrogen. One volume (relative to cells) of 2x lysis buffer, containing a protein inhibitor cocktail (2.8 µM leupeptin, 8 µM pepstatin A, 4 mM PMSF, 8 mM benzamidine, 8 µM antipain, 4 µM chymostatin in ethanol) and phosphatase inhibitors (2 mM sodium fluoride, 1.2 mM β-glycerophosphate, 0.04 µM sodium vanadate, 2 mM EGTA, 10 mM sodium pyrophosphate), was added. Cell extract was clarified by a low speed centrifugation (3,000 rpm) followed by additional centrifugation for 1 hour at 42000 rpm in a Beckman Sw55Ti rotor. The clarified crude extract (CCE) was adjusted to 10 mg/ml with 1x lysis buffer in the various immunoprecipitation experiments. 1 ml of CCE was precleared by incubation with 40 µl of 50% (v/v beads/1x lysis buffer) Protein G slurry (GE Healthcare) for 1 hour at 4ºC on a rotating wheel. Pre-cleared supernatants were incubated with either 20 µg of the anti-FLAG Mab M2 (Sigma), 20 µg of the anti-MYC Mab 9E11 (Abcam) or 20 µg of unspecific mouse IgG (Sigma). Samples were incubated for 2 hours at 4ºC on a rotating wheel and centrifuged at 14000 rpm for 15 minutes at 4ºC. 40 µl of 50% protein G slurry (GE Healthcare) were added to the supernatants, incubated on a rotating wheel for 2 hours at 4ºC and recovered by centrifugation. Immunoprecipitated samples were washed four times with 1 ml of lysis buffer containing protease and phosphatase inhibitors. Beads were finally resuspended in 40µl of 3x Laemmli buffer (IP), boiled for 5 minutes and released proteins were separated on 6.5% (80/1 acrylamide/bisacrylamide) SDS-PAGE gels. Rad9, Chk1-3FLAG and Dpb11-13MYC were analysed as described above.

**ATP-dependent Release Assay:**

Yeast crude cell extracts from G2/M-arrested and bleocin treated cells were prepared as described above. One ml of CCE at 10mg/ml were used to immunoprecipitate Rad9-9MYC with the anti-MYC monoclonal antibody (9E11 from Abcam) as described . The Rad9-9MYC beads were subjected to an ATP-dependent release assay as detailed , with the following modifications: beads were washed four times with 1 ml of 1x lysis buffer containing protease and phosphatase inhibitors (see above) and subsequently washed twice with 1 ml of kinase buffer (25mM HEPES pH7.5, 5mM EGTA, 15mM MgCl_2_, 15mM KAc, 1x protease and 1x phosphatase inhibitors). The beads were resuspended in 60µl of kinase buffer alone or kinase buffer containing either 10mM ATP (BioLabs) or non-hydrolysable γS-ATP (Sigma). After 45 minutes of incubation at 25^o^C, the elution fractions (containing released proteins) were carefully removed from the beads. Elutions and boiled beads were analysed on a 6.5%, 80/1 acrylamide/bisacrylamide SDS-PAGE gel. Rad9-9MYC, Chk1-3FLAG and Rad53 were analysed by Western blotting as described above.

**Rad9 dephosphorylation Assay:**

Yeast crude cell extracts from asynchronous W303 cells were prepared as described above and diluted to 1mg/ml total protein concentration. 500µg of total protein were subjected to immunoprecipitation as described above with the following modifications: Ten microliters of 50% (v/v beads/1x lysis buffer) Protein A slurry (GE Healthcare) were used to preclear the cell crude extracts and added to the supernatants after incubation with 2.5µg of NLO5 polyclonal antibody to immunoprecipitate Rad9. Immunoprecipitated samples were washed four times with 1 ml of lysis buffer containing protease inhibitors as above. Beads containing Rad9 were incubated in 1x λ-protein phosphatase buffer (New England Biolabs) and 2 mM MnCl_2_ (New England Biolabs) containing 200 U of protein phosphatase (New England Biolabs) for 20 minutes at 30°C. The phosphatase inhibitor sodium orthovanadate (Sigma) was added to one reaction at the final concentration of 2.5mM. Phosphatase reactions were stopped by adding 20µl of 3x Laemmli sample buffer (125 mM Tris–HCl pH 6.8, 4% SDS, 10% β-mercaptoethanol, 20% glycerol, 0.002% bromophenol blue) and incubation at 95°C for 5 minutes. Rad9 profile was analysed by western blot as described above.

**Cdc28 complexes assays:**

**Tandem Affinity purification of Cdc28 complexes**

The TAP method was applied for purification of Cdc28/Cyclins complexes (Clb5-TAP-Cdk1, Clb3-TAP-Cdk1 and Clb2-TAP-Cdk1) as described previously . All steps were performed at 4^o^C, unless otherwise stated. TAP-Clb purification was performed on 2-4 liters of yeast culture. Large Scale Native (LSN) yeast extracts were poured into a 50ml tube containing 200-400µl of pre-equilibrated IgG Sepharose 50% beads slurry (Amersham) and rotated 30 min at 4^o^C. The beads were centrifuged at 1000g for 3 min and were transferred to a 5 ml gravity flow column (Pierce). Once the bead bed was formed, the supernatant (lysate) was loaded over the column and the Flow Through (FT) fraction collected for western blot analysis. The beads were first washed with 30ml of IPP150 buffer (25mM HEPES-HCl pH8.0, 150mM NaCl, 0.1% NP-40) followed by a second wash with 10ml of TEV cleavage buffer (25mM HEPES-HCl pH8.0, 150mM NaCl, 0.1% NP-40, 0.5mM EDTA, 1mM DTT). The column was rotated for 15min at 23^o^C in 1ml TEV cleavage buffer. The column was drained and cleavage of the TEV site was performed by adding 1ml of TEV buffer containing 100 units of AcTEV protease (Invitrogen) and rotating the column for 30min at 23^o^C. The elution fraction was then recovered by gravity flow. 1mM MgAc, 1mM imidazole and 5mM CaCl_2_ was added to the 1ml elution fraction. This was then transferred into a new column containing 100-200µls of calmodulin binding beads (CBB, Stratagene) pre-equilibrated with 10ml of IPP150 calmodulin binding buffer (10mM β-mercaptoethanol, 25mM HEPES-HCl PH8.0, 150mM NaCl, 1mM MgAc, 1mM imidazole, 2mM CaCl_2_, 0.1% NP-40) and rotated for 1h at 4^o^C. The entire content of column was then poured into a 5 ml gravity flow column and the FT fraction collected for western blot analysis. The beads were washed with 30ml of IPP150 calmodulin binding buffer. Ten fractions of 200µls were then eluted with IPP150 calmodulin elution buffer (10mM β-mercaptoethanol, 25mM HEPES-HCl PH8.0, 150mM NaCl, 1mM MgAc, 1mM imidazole, 2mM EGTA, 0.1% NP-40). 10µls of each fraction and 40µls of FT samples were loaded onto a 12% SDS-PAGE gel and analysed by Silver and Coomassie staining. Peak fractions were pooled and 15µl aliquots were frozen down to be used in kinase assays.

Cdc28/3HA-Cln2 was purified according to published protocols using the 12CA5 Monoclonal antibody. Large Scale Native yeast extracts were prepared from 3L of Cdc28/3HA-Cln2 cell culture and gently rotated with 500µls of anti-HA beads, prepared as described ([McCusker et al., 2007](#_ENREF_51)), for 2 hr at 4°C. The beads were pelleted by brief centrifugation, washed twice with 15ml of lysis buffer (50mM HEPES pH7.4, 175mM KCl, 1mM EGTA, 1mM MgCl_2_, 0.45% Tween, 5% Glycerol) and then transferred to a 5 ml gravity flow column (Pierce). The column was washed with 5ml of lysis buffer and then with 1ml of elution buffer (50mM HEPES pH7.4, 150mM KCl, 1mM EGTA, 1mM MgCl2, 0.05% Tween, 5% Glycerol). The column was transferred to room temperature and 250µl of elution buffer containing 1mg/ml HA tripeptide (Sigma) was added. After 15 min incubation, the elution fraction was collected. This step was repeated for a total of 8 fractions. 10µl of each fraction was loaded onto a 12% SDS-PAGE gel and analysed by Silver and Coomassie staining. Peak fractions were pooled and 15µl aliquots were frozen down to be used in kinase assays. The concentration of purified kinase complexes was within the range of 0.1-1ng/µl.

**In vitro Cdc28 kinase assays:**

The quantitative phosphorylation assays of recombinant CAD were performed as previously described . Substrate concentrations were kept at 2 μM (in the linear [S] vs v0 range, several fold below the estimated KM value), and the initial velocity conditions were defined as an initial substrate turnover ranging up to 10% of the total turnover. This was estimated by a long-term incubation (30 and 60 minutes) with excess amounts of Cdc28/Cyclin (13nM) in the standard reaction mixture given below. Amounts of active Cdc28/Cyclin complexes to be used in the final experiment were normalized according to the specificity constants of the phosphorylation of H1, model substrate for Cdc28, by each Cdc28/Cyclin (k_cat_/K_M_ values are 1.394 for Cln2; 0.605 for Clb5; 2.237 for Clb3 and 4.9 for Clb2 4.9; Koivomagi et al. 2011).

The final adjusted kinase assay was performed in 20µl containing 2µM of H1 (Sigma), CAD^WT^ or CAD^CDK1-9A^ and about 0.1-1 nM of purified cyclin-Cdc28 complex. The basal composition of the assay mixture contained 50 mM Hepes pH 7.4, 100 mM NaCl, 0.2 mg/ml BSA, 1mM DTT, 500nM Cks1 (from M. Loog), 100 μM ATP and 0.5µCi ^32^P-γ-ATP (6000Ci/mmol, Perkin Elmer). The reactions were incubated at room temperature and reaction aliquots were taken at two time points (8 and 16 min, the latest is shown in Figure 2B). The reactions were stopped by adding 4x SDS-PAGE sample buffer and the totality loaded on a 12% SDS-PAGE gel. The gel was Coomassie-stained and air-dried overnight.

The kinase assay on the peptide array was performed as reported before with some modifications.. The array was bathed in 100% ethanol for 5 min, washed twice in 25ml of Kinase buffer containing 1mg/ml BSA and incubated overnight in 25ml of the same buffer. The array was then incubated with kinase buffer containing 39ng of Clb2-Cdc28, 0.2mg/ml BSA, 500µM ATP and 10µCi ^32^P-γ-ATP (Perkin Elmer) and the reaction was allowed to occur for 1hr at room temperature. The reaction on the array was then stopped by adding an excess of 1% SDS and incubated for 1hr at 65C. The array was incubated overnight with 0.5% phosphoric acid, washed three times in the same solution, and once in 50%, 25%, 12.5% and 6.25% methanol. The array was then rinsed in 96% ethanol, air dried and exposed.

Radiolabel incorporation into dried gels or peptide array membranes was visualized either by autoradiography or using a phosphorimager. For autoradiography, gels were exposed for the appropriate time to X-ray film (Kodak) in a film cassette at -80°C. For phosphorimager analysis, gels were exposed to a phosphoscreen (FUJIFilm) for time ranging from 30 minutes to 16 hours before analysis using a FUJIFilm 5100 FLA phosphorimager. When necessary, phosphorimager results were further quantified using Image Gauge software.

**Rad9 peptides pull down experiments**

**Chk1-3FLAG purification:**

For each pull down, Chk1 was initially semi-purified from 15mg of pre-cleared native protein extract prepared as described above from *rad9Δ Chk1-3FLAG* cells. The immunoprecipitation was performed using 100µl of pre-equilibrated M2 FLAG-affinity gel (Sigma, A2220) and allowed to occur for 2 hours, on a rotating wheel at 4°C. The supernatant (S/N) was then kept, the beads were washed four times with 1x lysis buffer containing only protease inhibitors and resuspended in 100µl of this buffer. Chk1-3FLAG was eluted by adding 6µl of 3xFLAG peptide (5mg/ml, Sigma) and incubated for 1 hour on a rotating wheel at 4°C. Elutions were pooled before the subsequent step. The efficiency of Chk1-3FLAG purification and elution was verified by loading a comparable amount of the initial cell crude extract (CCE), of the S/N_1_, as well as of Chk1 elution and FLAG M2 beads onto a 6.5% 80/1 acrylamide/bis-acrylamide SDS-PAGE gel to visualize Chk1-3FLAG by western blotting.

**Pull down experiments:**

Biotin-labelled Rad9 peptides, synthesised by Pepceuticals Limited (Leicestershire, UK), were used in 100 molar excess to saturate 80μl of pre-washed Streptavidin Dynabeads M-280 (Invitrogen). In experiments involving phosphatase treatment, peptides-beads were treated with 10 units of λ a  n  for 20min at 30°C in the presence or in the absence of phosphatase inhibitors, followed by 4 washes with 1x Lysis buffer. Eluted Chk1-3FLAG (±100µls) was added to the peptides beads and incubated for 2 hours, on a rotating wheel at 4°C. The beads were washed 4 times with 1x lysis buffer containing protease inhibitors and resuspended in 40µl of 3x Sample Buffer. Samples were boiled at 95°C for 5min, 5µl of each sample was loaded onto a 20% SDS-PAGE gel for peptide detection by Ponceau staining and what remained of the pull down sample was loaded onto a 6.5% 80/1 acrylamide/bis-acrylamide SDS-PAGE gel to visualize Chk1-3FLAG.

**Rad9 mass spectrometry analyses**

**Rad9 purification:**

Cell cycle-modified (C-Rad9) Rad9-GFP-FLAG was purified by double immunoaffinity purification from whole-cell extracts, prepared from 10L of asynchronously growing, G1 or G2/M arrested cells (1-2x10^7^ cells/ml) as described above, with minor modifications (lysis buffer contained no EDTA and 0.1% CHAPS instead of 0.1% Tween20 and 0.01% NP-40). The efficiency of the G1 and G2/M arrests using 10μg/ml α-factor and 20μg/ml nocodazole respectively was verified by microscopy. Approximately 1.5g of total protein was obtained for each crude cell extract. Extracts were precleared with 200μl of packed protein G beads (GE Healthcare) prior to incubation with 200μl of packed FLAG M2-affinity beads (Sigma-Aldrich) for 2h at 4^o^C. Four successive elutions were performed using 200μl of 3xFLAG peptide (100μg/ml, Sigma-Aldrich) and Rad9-GFP-FLAG-containing fractions were identified by western blotting. 20μl of GFP-Trap agarose beads (Chromotek) was added to the pooled Rad9-GFP-FLAG containing fractions. Batch binding was allowed to occur for 2h at 4^o^C with gentle agitation. The beads were washed 3 times with wash buffer (150mM KAc, 50mM HEPES pH7.5) and once with ultra pure water. Washed beads were resuspended in an equal volume of 0.1% glacial acetic acid. The presence of purified Rad9-GFP-FLAG was verified by silver stain gel on which 1μl of the final beads was loaded (Figure S7A).

**Mass Spectrometric Analysis of Rad9-GFP-FLAG:**

Rad9-GFP-FLAG purified from asynchronous, G1- or G2/M-arrested cells was reduced and carbamidomethylated on GFP beads at room temperature using dithiothreitol (Sigma Aldrich, St. Louis, MO) and iodoacetamide (Sigma Aldrich), respectively, then subjected to proteolytic digestion (1:20 enzyme to substrate) using endoproteinase Lys-C (Roche, Penzberg, Germany) using methods similar to those previously reported (Hall et al 2010). Half of each sample was subjected to sub-digestion using trypsin (Promega, Madison, WI). Prior to mass spectrometric analysis, each digest was dried down and resuspended in 0.1% acetic acid in water. For each analysis, a 1 pmol fraction was pressure loaded onto a 360 μm o.d. × 75 μm i.d. fused silica capillary precolumn packed with 5 cm of C18 reverse-phase resin (5–20 μm diameter, 120 Å pore size, YMC Co., Ltd., Kyoto, Japan). Following a 15 min desalting rinse using 0.1 M acetic acid, the precolumn was connected to a 360 μm o.d. × 50 μm i.d. analytical column packed with 6-8 cm of C18 resin (5 μm diameter, 120 Å pore size, YMC Co., Ltd.) and equipped with an electrospray emitter tip as previously described (Udeshi et al 2008). Endoproteinase Lys-C or trypsin generated peptides were then gradient eluted into the mass spectrometer using electrospray ionization at a flow rate of 60 nL/min using an LC gradient previously described (Syka et al 2004). Mass analysis was completed using an acquisition method consisting of one high resolution MS1 scan (resolving power of 50,000 (FT Ultra) or 60,000 (Orbitrap) at m/z 400) acquired in the FT-ICR or Orbitrap followed by 5 data dependent MS2 scans using collisionally-activated dissociation (CAD) and electron transfer dissociation (ETD) acquired in the ion trap of the LTQ-FT Ultra or LTQ-Orbitrap hybrid instrument (Thermo Fisher Scientific, Bremen, Germany). Data dependent parameters included a repeat count of 3, repeat duration of 30 s, and exclusion list duration of 30 s. MS2 parameters for ETD scans included 35 ms reaction time, 3 m/z precursor isolation window, charge state rejection “on” for +1 and unassigned charge state precursor ions, 5× 10^5^ FTMS (Orbitrap) or 1× 10^6^ (FT Ultra) full automated gain control target, 1 × 10^4^ ITMS^n^ automated gain control target, and 2 × 10^5^ reagent target with azulene as the electron transfer reagent.

**Supplemental References**

Clerici, M., Baldo, V., Mantiero, D., Lottersberger, F., Lucchini, G., and Longhese, M.P. (2004). A Tel1/MRX-dependent checkpoint inhibits the metaphase-to-anaphase transition after UV irradiation in the absence of Mec1. Mol Cell Biol *24*, 10126-10144.

Cocker, J.H., Piatti, S., Santocanale, C., Nasmyth, K., and Diffley, J.F. (1996). An essential role for the Cdc6 protein in forming the pre-replicative complexes of budding yeast. Nature *379*, 180-182.

Diani, L., Colombelli, C., Nachimuthu, B.T., Donnianni, R., Plevani, P., Muzi-Falconi, M., and Pellicioli, A. (2009). Saccharomyces CDK1 phosphorylates Rad53 kinase in metaphase, influencing cellular morphogenesis. J Biol Chem *284*, 32627-32634.

Gelbart, M.E., Rechsteiner, T., Richmond, T.J., and Tsukiyama, T. (2001). Interactions of Isw2 chromatin remodeling complex with nucleosomal arrays: analyses using recombinant yeast histones and immobilized templates. Mol Cell Biol *21*, 2098-2106.

Gilbert, C.S., Green, C.M., and Lowndes, N.F. (2001). Budding yeast Rad9 is an ATP-dependent Rad53 activating machine. Mol Cell *8*, 129-136.

Gyuris, J., Golemis, E., Chertkov, H., and Brent, R. (1993). Cdi1, a human G1 and S phase protein phosphatase that associates with Cdk2. Cell *75*, 791-803.

Hall, E.H., Balsbaugh, J.L., Rose, K.L., Shabanowitz, J., Hunt, D.F., Brautigan, D.L. (2010) Comprehensive analysis of phosphorylation sites in tensin1 reveals regulation by p38MAPK. Mol Cell Prot 9, 2853-2863.

Koivomagi, M., Valk, E., Venta, R., Iofik, A., Lepiku, M., Morgan, D.O., and Loog, M. (2011). Dynamics of Cdk1 substrate specificity during the cell cycle. Mol Cell *42*, 610-623.

Kushnirov, V.V. (2000). Rapid and reliable protein extraction from yeast. Yeast *16*, 857-860.

Longtine, MS., McKenzie A 3^rd^., Demarini, DJ., Shah NG., Wach, A., Brachat, A., Philippsen, P., Pringle, JR. (1998). Additional modules for versatile and economical PCR-based gene deletion and modification in Saccharomyces cerevisiae. Yeast *14*, 953-61.

McCusker, D., Denison, C., Anderson, S., Egelhofer, T.A., Yates, J.R., 3rd, Gygi, S.P., and Kellogg, D.R. (2007). Cdk1 coordinates cell-surface growth with the cell cycle. Nat Cell Biol *9*, 506-515.

Puig, O., Caspary, F., Rigaut, G., Rutz, B., Bouveret, E., Bragado-Nilsson, E., Wilm, M., and Seraphin, B. (2001). The tandem affinity purification (TAP) method: a general procedure of protein complex purification. Methods *24*, 218-229.

Shirayama, M., Zachariae, W., Ciosk, R., and Nasmyth, K. (1998). The Polo-like kinase Cdc5p and the WD-repeat protein Cdc20p/fizzy are regulators and substrates of the anaphase promoting complex in Saccharomyces cerevisiae. EMBO J *17*, 1336-1349.

Syka J. E., Coon J. J., Schroeder M. J., Shabanowitz J., Hunt D. F. (2004) Peptide and protein sequence analysis by electron transfer dissociation mass spectrometry. Proc Natl Acad Sci U S A 101, 9528–9533.

Tegge, W.J., and Frank, R. (1998). Analysis of protein kinase substrate specificity by the use of peptide libraries on cellulose paper (SPOT-method). Methods Mol Biol *87*, 99-106.

Ubersax, J.A., Woodbury, E.L., Quang, P.N., Paraz, M., Blethrow, J.D., Shah, K., Shokat, K.M., and Morgan, D.O. (2003). Targets of the cyclin-dependent kinase Cdk1. Nature *425*, 859-864.

Udeshi N. D., Compton P. D., Shabanowitz J., Hunt D. F., Rose K. L. (2008) Methods for analyzing peptides and proteins on a chromatographic timescale by electron-transfer dissociation mass spectrometry. Nat Protoc 3, 1709–1717.
